# A multi-omics analysis reveals the unfolded protein response regulon and stress-induced resistance to folate-based antimetabolites

**DOI:** 10.1038/s41467-020-16747-y

**Published:** 2020-06-10

**Authors:** Stefan Reich, Chi D. L. Nguyen, Canan Has, Sascha Steltgens, Himanshu Soni, Cristina Coman, Moritz Freyberg, Anna Bichler, Nicole Seifert, Dominik Conrad, Christiane B. Knobbe-Thomsen, Björn Tews, Grischa Toedt, Robert Ahrends, Jan Medenbach

**Affiliations:** 1 Biochemistry I, University of Regensburg, Regensburg, Germany; 2 Leibniz-Institut für Analytische Wissenschaften—ISAS—e.V., Dortmund, Germany; 3 Institute for Clinical Chemistry and Laboratory Medicine, Technical University Dresden, Dresden, Germany; 4 Department of Neuropathology, Heinrich Heine University Düsseldorf, Düsseldorf, Germany; 5 Molecular Mechanisms of Tumor Invasion, German Cancer Research Center (DKFZ), Heidelberg, Germany; 6 Department of Analytical Chemistry, University of Vienna, Vienna, Austria; 7 Computational Biology Unit, European Molecular Biology Laboratory (EMBL), Heidelberg, Germany; 8 Present Address: AbbVie Deutschland GmbH & Co.KG, Wiesbaden, Germany

**Keywords:** RNA, Reverse transcription polymerase chain reaction, Protein aggregation, Gene expression, Gene regulation

## Abstract

Stress response pathways are critical for cellular homeostasis, promoting survival through adaptive changes in gene expression and metabolism. They play key roles in numerous diseases and are implicated in cancer progression and chemoresistance. However, the underlying mechanisms are only poorly understood. We have employed a multi-omics approach to monitor changes to gene expression after induction of a stress response pathway, the unfolded protein response (UPR), probing in parallel the transcriptome, the proteome, and changes to translation. Stringent filtering reveals the induction of 267 genes, many of which have not previously been implicated in stress response pathways. We experimentally demonstrate that UPR‐mediated translational control induces the expression of enzymes involved in a pathway that diverts intermediate metabolites from glycolysis to fuel mitochondrial one‐carbon metabolism. Concomitantly, the cells become resistant to the folate-based antimetabolites Methotrexate and Pemetrexed, establishing a direct link between UPR‐driven changes to gene expression and resistance to pharmacological treatment.

## Abstract (web summary)

The unfolded protein response (UPR) is a stress response pathway implicated in numerous diseases and chemotherapy resistance. Here, the authors define the UPR regulon with a multi-omics strategy, uncovering changes to mitochondrial one-carbon metabolism and concomitant resistance to folate-based therapeutics.

## Introduction

Tumor cells can escape apoptotic cell death, and survive and proliferate in hostile environments often characterized by a lack of nutrients and oxygen. For this, cancer cells exploit intrinsic adaptive mechanisms and stress response pathways such as the endoplasmic reticulum (ER) unfolded protein response (UPR). The UPR is triggered by the accumulation of unfolded or misfolded proteins in the ER and it aims at reinstating cellular homeostasis, or, if that fails, at triggering of apoptosis. It is of clinical importance and has key roles in a variety of disorders, including metabolic diseases, neurodegenerative disorders, and inflammatory processes^1–5^. Moreover, the UPR is broadly implicated in cancer progression and malignancy, survival and proliferation of cancer cells, and their resistance to pharmacological treatment^6–13^. In particular, the latter is a major predictor of mortality in cancer patients.

Given the central role of the UPR in clinically important cell fate decisions, it has emerged as an attractive target for therapeutic intervention with the aim to tilt the balance of protective effects versus apoptosis for the benefit of patients^14–22^. Despite its clinical relevance, it is still only poorly understood how the UPR contributes to chemoresistance of cancer cells and a better understanding of the underlying mechanisms and pathways is urgently required to improve the therapeutic outcome.

In general, the UPR utilizes three main branches to sense perturbations in ER homeostasis and to trigger the appropriate cellular responses^23^. These depend on three proteins that span the membrane of the ER to probe its status: inositol-requiring enzyme-1 alpha (IRE1*α*, aka ERN1), activating transcription factor 6 (ATF6), and PKR-like endoplasmic reticulum kinase (PERK, aka EIF2AK3). The first two proteins with their respective signal transduction pathways mainly exert their function via transcriptional reprogramming, in contrast PERK controls cellular translation through phosphorylation of eukaryotic translation initiation factor 2 alpha (EIF2S1)^24^.

To gain a comprehensive and systems-wide understanding of the UPR, we have pursued a multi-omics approach that, upon chemical induction of the UPR with two different compounds, monitors in parallel several parameters in the astrocytoma cell line LN-308. Changes to the cellular transcriptome (by high throughput sequencing) and proteome (by shotgun and targeted proteomics) as well as altered translation status (by ribosome profiling) were measured after different time points of treatment. Integration of the datasets reveals the induction of 267 genes (the UPR regulon), including numerous cancer-relevant factors and metabolic enzymes. We detect evidence for a UPR-dependent metabolic reprogramming that diverts metabolites from glycolysis to mitochondrial one-carbon (1C) metabolism. This renders the cells insensitive to treatment with the FDA-approved folate-based antimetabolites Methotrexate and Pemetrexed, establishing a direct link between UPR-driven changes to gene expression and resistance to pharmacological treatment.

## Results and discussion

### Induction of the UPR in LN-308 cells

To trigger the UPR in the astrocytoma-derived cell line LN-308, we used two different compounds: the nucleoside antibiotic and *N*-glycosylation inhibitor tunicamycin (TM) and the SERCA inhibitor thapsigargin (TH)^25,26^. We determined concentrations for both compounds that result in the activation of key factors of the UPR and/or their effects on downstream targets while not causing a full replicative arrest of the cells (200 nM Thapsigargin or 2.5 µg/ml Tunicamycin). Treatment of LN-308 cells under these conditions rapidly induces IRE*α*-mediated, cytoplasmic processing of X-box Binding Protein 1 (XBP1) mRNA, which can be detected by RT-PCR after 2 h of treatment (Supplementary Fig. 1A). This results in the production of functional, full-length XBP1s protein (Supplementary Fig. 1B), which can only be translated from the fully processed mRNA. The ratio of spliced versus unspliced XBP1 mRNA oscillates over the time-course of the treatment which is, to some extent, also reflected in changes to the XBP1s protein level, underscoring the dynamic and adaptive nature of the UPR. Another rapid response to ER stress is phosphorylation and activation of PERK, which results in a slightly reduced gel mobility^27^ that can be detected already after 1 h of treatment and which becomes more pronounced at later times (Supplementary Fig. 1B). Concomitantly, increased phosphorylation of the PERK downstream target EIF2S1 on Ser51 can be observed (Supplementary Fig. 1B). This in turn promotes activating transcription factor 4 (ATF4) protein production by a translation re-initiation mechanism that is sensitive to EIF2S1 phosphorylation^28,29^. ATF4 protein levels are elevated at 2 h and peak at 4–6 h, however, decreased protein levels are observed at later time points (e.g. after 24 h of treatment). In contrast, a different kinetic can be observed for the induction of the ER chaperone heat shock protein family A (Hsp70) member 5 (HSPA5 aka BiP): an increase in abundance of the protein can be detected only after 4–6 h, however, the protein abundance increases continuously during the treatment, resulting in a strong accumulation of HSPA5 protein after 24 h (Supplementary Fig. 1B).

### Staged sampling of the UPR

**Fig. 1:**
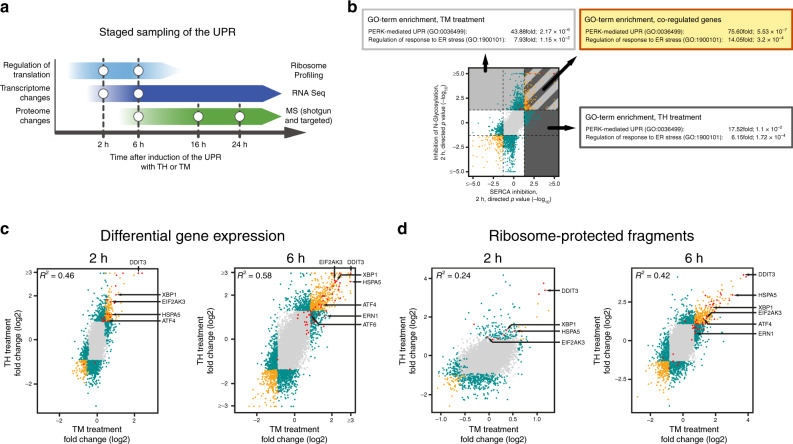
A multi-omics approach to analyze the UPR. **a** Schematic depiction of the staged sampling scheme employed in this study. **b** Comparison of TM- and TH-induced changes to RNA expression levels highly enriches for factors involved in the UPR. Plotted are directional *P*-values of differentially expressed protein-coding genes after 2 h of treatment. Enrichment scores and *P*-values for the GO-terms PERK-mediated UPR (GO:0036499) and regulation of response to ER stress (GO:1900101) are given for genes that are upregulated after (1) inhibition of N-glycosylation (by TM treatment, area shaded in light gray) (2) after inhibition of the SERCA (by TH treatment, area shaded in dark gray) and (3) upregulated in both treatments (striated area). **c** Comparison of differences in mRNA abundance of protein-coding genes induced by treatment with TM or TH for 2 h (left panel) and 6 h (right panel) (see also Supplementary Fig. 2 and Supplementary Data 2). **d** Comparison of TM- and TH-induced changes to ribosome-protected fragment counts after 2 h (left panel) and 6 h of treatment (right panel) (see also Supplementary Fig. 4 and Supplementary Data 3). In **c**, **d** log2-fold changes of mean values are plotted. Two criteria were used to identify regulated genes: (a) fold-change in the 5% (downregulation) and 95% (upregulation) quantiles and (b) a *P*-value threshold (<0.05). Color coding **b**–**d**—gray dots: no significant change in either treatment; green dots: significantly upregulated or downregulated in only one treatment or inversely regulated upon TM and TH treatment; yellow dots: statistically significant regulation in the same direction in both treatments; red dots: known factors of the UPR. Select factors involved in the UPR are marked by arrows.

To gain a more comprehensive insight into the adaptive response initiated by ER stress, we expanded our analyses and employed a multi-omics approach to probe cellular translation (by ribosome profiling), the transcriptome, and the proteome at different time points. As changes to the translation status of the cell are rapid (e.g. ATF4 induction after phosphorylation of EIF2S1, Supplementary Fig. 1B), we performed ribosome profiling at early time points, after 2 and 6 h of treatment (Fig. 1a). In parallel, we assayed the cellular transcriptome as a reference for the determination of translation efficiencies of different RNA species. In addition, the transcriptional profiling data yield insight into (early) RNA processing events (such a XBP1 mRNA splicing, Supplementary Fig. 1A), but also show UPR-dependent transcriptional reprogramming towards later time points when changes to the steady state levels of an increasing number of mRNAs become detectable. Finally, proteomic changes were analyzed at 6 h and later time points (16 h and 24 h) to cover early translational responses but also to gain insight into adaptive changes to continuously ongoing and developing chronic ER stress—a situation frequently encountered in disease.

**Fig. 2:**
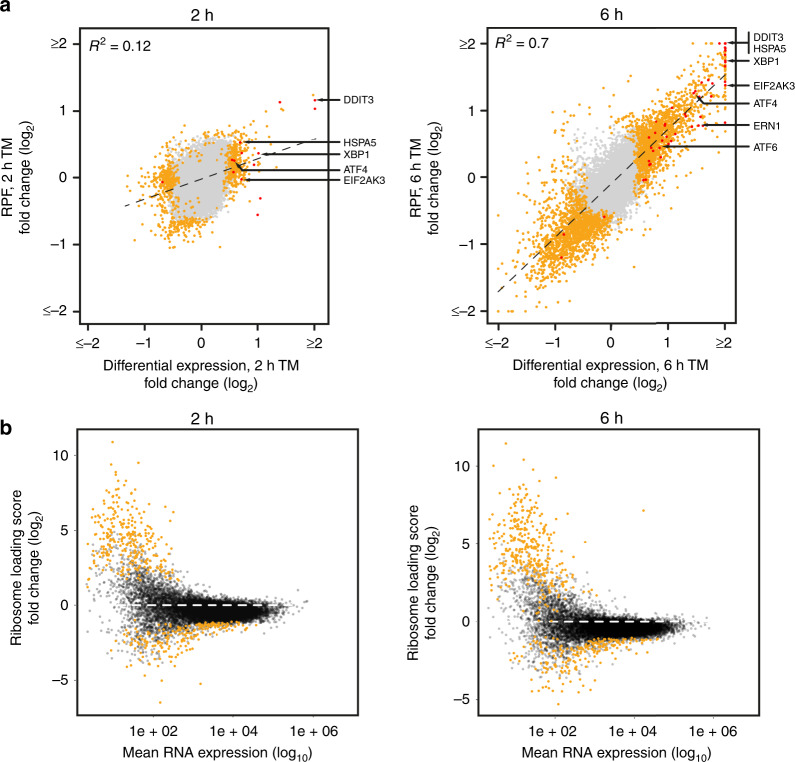
Global analysis of UPR-mediated translation regulation. **a** Comparison of differential RNA expression and changes to RPF abundance of protein-coding RNAs after 2 h (left panel) or 6 h (right panel) of TM treatment. Loci with a *P*-value below threshold are depicted yellow, plotted are log2-fold changes of mean values. **b** Analyses of ribosome loading scores of protein-coding RNAs after 2 h (left panel) and 6 h (right panel) of TM stimulation. Note the overall reduction in ribosome loading (for reference the dashed line indicates no change in ribosome loading). RNAs that exhibit statistically significant changes in ribosome loading are shown in yellow.

**Fig. 3:**
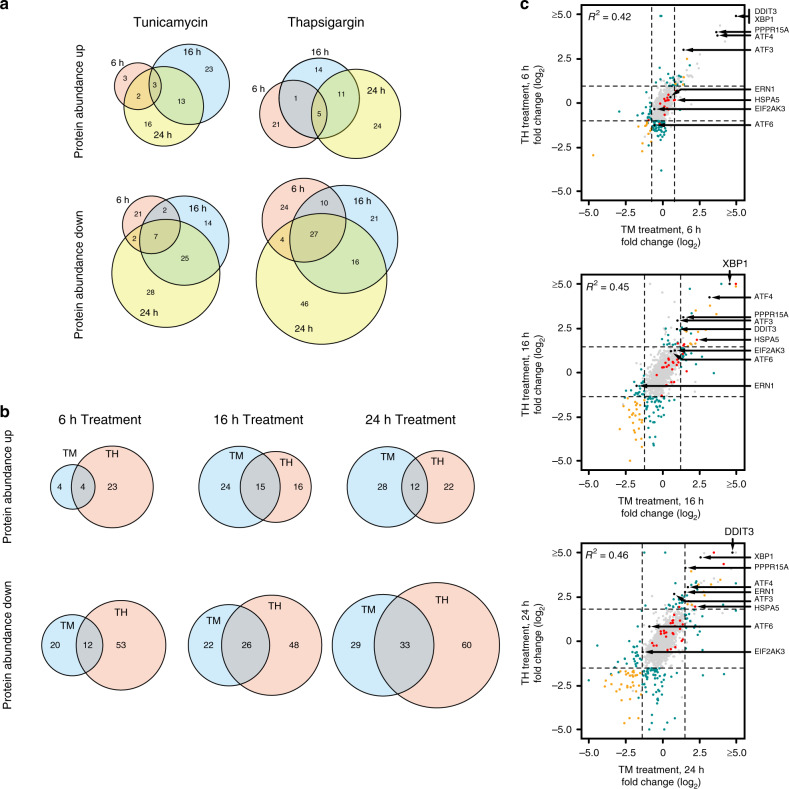
UPR-induced changes to the cellular proteome. **a** Time-resolved comparison of changes to protein abundance after TM treatment (left) or TH treatment (right). **b** Comparison of TM and TH-induced changes to protein abundance after 6, 16, and 24 h of treatment. **c** Comparison of mean values of protein abundance changes 6 h (top panel), 16 h (middle panel), and 24 h (bottom panel) after stimulation with either TH or TM. Data from shotgun proteomics are color-coded as in Fig. 1. Additional data points from a targeted proteomics approach^30^ are shown as black dots. Select proteins involved in the UPR are marked by arrows. (see also Supplementary Fig. 5 and Supplementary Data 4). Two criteria were used to filter for regulated genes: a fold-change median ± 2 * standard deviation (as indicated by the dashed lines), and a *P*-value threshold (<0.05).

Our findings attest to the validity of this approach: (1) our analyses reveal significant and wide-spread changes of the cellular transcriptome and altered translation after 2 and 6 h of treatment (Figs. 1, 2, Supplementary Figs. 2, 3)—in contrast, at 6 h the cellular proteome remains almost unchanged and adaptive changes to the steady state levels of numerous proteins become apparent only at later stages (Fig. 3), and (2) as expected, for most responsive genes transcriptomic changes precede changes in protein abundance. This is also reflected by the finding that transcriptomic changes after 6 h enrich for the same GO-terms as proteomic changes after 16 h. However, no such correlation can be found when comparing the 6 h time points.

### Differential gene expression during the UPR

To experimentally assess UPR-mediated transcriptional reprogramming, we analyzed RNA expression by high throughput sequencing after 2 and 6 h of treatment with either TM or TH (Supplementary Data 2). Both compounds cause significant changes to the cellular transcriptome after 2 h of treatment.

The steady state levels of ∼700 protein-coding RNAs respond to inhibition of N-glycosylation by TM for 2 h (315 with increased abundance, 381 with decreased abundance, Supplementary Fig. 2A), and after 6 h of treatment we find ∼1500 mRNAs that change abundance (793 with increased abundance, 794 with decreased abundance). Importantly, there is only a limited overlap between both time points (*R*^2^ = 0.32, Supplementary Fig. 2B), and only few transcripts are upregulated or downregulated at both 2 and 6 h of TM treatment (110 up and 171 down, Supplementary Fig. 2C) indicating that the cellular response to TM treatment is dynamic and changes from 2 to 6 h of treatment.

Exposure to TH induces an even greater change to the cellular transcriptome: after 2 and 6 h, we detect significant changes to the abundance of 1442 and 1438 mRNAs, respectively (Supplementary Fig. 2A). The changes to the cellular transcriptome at 2 and 6 h correlate better (*R*^2^ = 0.71, Supplementary Fig. 2B) than observed for the treatment with TM. This is also reflected in the much higher number of RNAs that show a similar regulation after 2 and 6 h of TH treatment: ∼30% of the transcripts that are upregulated after 2 h also show an increased abundance after 6 h (Supplementary Fig. 2C). The overlap is even more pronounced for the transcripts that exhibit reduced steady state levels upon treatment (∼43% of the transcripts that show reduced levels at 2 h of treatment are still downregulated after 6 h of treatment).

### UPR-mediated translational reprogramming

Phosphorylation of EIF2S1 by PERK upon accumulation of unfolded proteins in the ER rapidly attenuates translation of the vast majority of messenger RNAs. In agreement with this, we observe an ~34% reduction of translation after 6 h of TM treatment as evidenced by metabolic incorporation of ^35^S-labeled methionine and a decrease of polysomal complexes (Supplementary Fig. 3). However, translation of the major coding region of a group of transcripts is insensitive to EIF2S1 phosphorylation and increased translation can be observed under stress conditions. This is thought to mostly depend on upstream open reading frames (uORFs) and often involves translation re-initiation^28^.

To better understand UPR-mediated control of translation during ER stress, we employed ribosomal profiling, a quantitative and transcriptome-wide systems analysis of translation. After limited ribonucleolytic digestion, mRNA-derived ribosome-protected fragments (RPFs) are subjected to high throughput sequencing, revealing with high resolution the positions of (translating) ribosomes on mRNAs.

Our analyses reveal wide-spread changes to the abundance of RPFs upon treatment with either TM or TH (Supplementary Data 3). In agreement with attenuated translation due to phosphorylation of EIF2S1, we find in particular at early time points (2 h after induction of the UPR) an overall reduction of RPFs from numerous loci, (TM 171, TH 386). In contrast, under the same conditions, only few protein-coding genes yield more RPFs (TM 74, TH 155) (Supplementary Fig. 4A). After 6 h of continuous treatment, the effect is less pronounced and the numbers of loci that yield significantly more RPFs or that generate fewer reads are comparable. Again, we only detect a poor correlation between the genes that are upregulated or downregulated upon treatment with TM or TH after 2 and 6 h (Supplementary Fig. 4B) suggesting that ER stress-induced gene regulation is highly dynamic.

Changes to the abundance of RPFs from a protein-coding locus can be essentially caused by either, a change in mRNA abundance, an altered translation rate, or both. As such, ribosome profiling, when not corrected for changes in RNA abundance, monitors both transcriptome changes and regulated translation. After 2 h of TM treatment, the detected changes to RNA abundance and RPFs exhibit little correlation (*R*^2^ = 0.12, Fig. 2). This suggests that either regulation of translation and transcription affects different gene sets, or that effects on translation dominate over mRNA abundance changes at this early time point. In contrast, after 6 h a much higher correlation can be observed between changes to RPF counts and RNA abundance (*R*^2^ = 0.7, Fig. 2). This indicates that after prolonged treatment most changes to gene expression are driven by altered RNA steady state levels instead of selective translation, which suggests that at least at this time-point transcriptional regulation dominates over translational control.

To get further insight into translational control under these cellular stress conditions, we approximated mRNA translation efficiency by the calculation of ribosome loading scores (RPF counts normalized by RNA abundance). Changes to ribosome loading of individual transcripts are indicative of changes to translation rates as they reflect either an altered density of translating ribosomes or, less frequently, ribosome stalling. In our data, we observe an overall decrease in ribosome loading after treatment (Fig. 2b and Supplementary Fig. 3B), which agrees with an overall decrease of translation upon PERK-mediated phosphorylation of EIF2S1 at serine 51 that is initiated as an immediate response to ER stress (Supplementary Fig. 1B). In agreement with previous studies of translation rates under stress conditions^31,32^, we find numerous transcripts that appear to resist EIF2 phosphorylation and exhibit increased association with ribosomes upon induction of the UPR (Fig. 2b). We also detect a set of mRNAs that is hyper-sensitive to EIF2S1 phosphorylation and which produces significantly fewer RPFs under stress conditions than the average mRNA (Fig. 2b).

### Proteomic changes during the UPR

To globally assess the steady state changes of the proteome, we performed shotgun MS analyses at three different time points after treatment with either TM or TH. We detect 4131 proteins with at least two peptides of which ~50% (2136 proteins) exhibit significant abundance changes in one of the experimental conditions (Supplementary Data 4). Of these proteins, 309 exhibit an abundance change of ±2-fold the standard deviation (7% of the proteome). As predicted, changes to the proteome appear with a delay compared to the changes observed for translation rates and RNA abundance. In agreement with this, the abundance of only few proteins is changed after 6 h of treatment (TM 40, TH 92), whereas the number of regulated proteins increases at later time points (TM 102, TH 127 after 24 h, Fig. 3). Overall, more proteins are found to be downregulated than upregulated, which is in good agreement with the ribosome profiling data and indicative of a globally reduced protein synthesis rate (Fig. 2). Again, we detect a limited overlap between the proteins that change abundance after treatment with either TH or TM (20% overlap for upregulated and 24% for downregulated genes, Fig. 3). Most of the proteins involved in UPR-mediated stress signaling are not abundantly expressed, hampering their detection by shotgun proteomics. We previously established a targeted proteomics workflow to monitor the expression of altogether 8 factors known to have key roles in the UPR (IRE1, PERK, ATF6, XBP1, GADD34, CHOP, ATF4, and ATF3)^30^. Using this approach, we confirm a ∼30-fold induction of ATF4 protein 6 h after treatment with either TM or TH. Similarly, we detect a ∼60-fold increase in XBP1 and a ∼50-fold increase in CHOP protein after induction of the UPR. This is consistent with our analyses of translation rates, RNA-abundance and protein levels by western blotting (Figs. 1–3, Supplementary Fig. 1).

### Co-regulation by TM and TH treatment

Importantly, perturbations of calcium homeostasis (by treatment with TH) or protein glycosylation (by treatment with TM) also induce adaptive cellular responses other than the UPR (side effects)^25,26^, resulting in gene expression changes unrelated to accumulation of unfolded proteins in the ER. As TM and TH interfere with very different cellular pathways, we expect on the one hand distinct, cellular responses that differ between the two reagents. On the other hand, we reasoned that adaptive changes to gene expression that are shared between the two chemical treatments are very likely driven by the UPR. Hence, filtering for gene expression changes observed in both treatments would allow us to specifically enrich for UPR-mediated cellular responses (Fig. 1b).

Once triggered, the UPR exhibits a characteristic order of events whose timing is largely independent of the nature of the stimulus. Rather it is determined by the enzymatic properties of the regulatory factors and the kinetics of the gene expression program. This is illustrated by the fact that, despite being the much stronger stimulus that results in much higher gene expression changes, TH-induced and UPR-driven changes to gene expression follow the same kinetics (and fluctuations) as the UPR-driven response induced by TM (Supplementary Fig. 1). We therefore independently analyzed changes to gene expression (RNA abundance and ribosome profiling) after 2 and 6 h to identify transcripts that are co-regulated in both treatment regimens at the same time point (Fig. 1c, d, Supplementary Figs. 2, 4, panels D, E).

As expected, the overall correlation between transcriptomic changes in both treatments is not very high (*R*^2^ = 0.46 after 2 h and *R*^2^ = 0.58 after 6 h of treatment, Fig. 1c and Supplementary Fig. 2E), confirming that TH and TM trigger different and only partially overlapping cellular responses. At the early time point, after 2 h of treatment, only few mRNAs exhibit a similar regulation in both treatment regimens (161 up, 109 down), whereas at 6 h, a larger proportion of RNAs is co-regulated (454 up, 232 down) (Supplementary Fig. 2D).

Previously, it has been reported that in cultured mouse embryonic fibroblasts (MEFs) the activation of IRE1 destabilizes ~120 different mRNAs through regulated IRE1-dependent decay (RIDD). RIDD preferentially targets ER-localized RNAs and initiates degradation by sequence-specific endonucleolytic cleavage, analogous to its role in XBP1 mRNA splicing^33^. In contrast to the strong RIDD-mediated regulation observed in *Drosophila*^34^, in MEFs the steady state level changes were generally smaller (log2-fold-change <2) and therefore challenging to detect^35^. We find that treatment with TM or TH results in the downregulation of largely distinct sets of mRNAs (∼10% co-regulated transcripts after 2 h of treatment, ∼20% after 6 h, Fig. 1c, Supplementary Fig. 2D, E) and after 2 or 6 h of treatment only 109 and 232 transcripts are co-down regulated in both treatments. Among these, we detect an enrichment of RNAs that encode membrane proteins. Strikingly, we do not detect an enrichment of previously identified RIDD target mRNAs among these RNAs, although the effective removal of the critical intron in the XBP1 mRNA indicates activation of IRE1*α* (Supplementary Fig. 1A). This suggests that under the conditions tested here the contribution of RIDD to RNA abundance changes is rather small and the decay of RIDD target RNAs is difficult to detect.

For the transcripts that are increased in abundance upon TH or TM treatment after 2 h, about 19% (161 protein-coding loci) are detected in both treatment conditions; after 6 h, this fraction is increased to ∼43% (454 protein-coding loci, Supplementary Fig. 2D). We detect many mRNAs encoding proteins involved in the UPR among the upregulated transcripts (Fig. 1c and Supplementary Fig. 2E, highlighted in red), many of which are co-regulated in both treatment regimens. In particular, under almost all conditions tested, we detect upregulation of transcripts that encode the major transducers of the UPR and their prominent targets: PERK (EIF2AK3), IRE1*α* (ERN1), XBP1, ATF6, ATF4, CHOP (DDIT3), and BiP (HSPA5) (Fig. 1c and Supplementary Fig. 2E, marked with arrows). For a more comprehensive analysis, we tested for the enrichment of GO-terms in the subsets of transcripts with increased abundance after 2 h. When individually analyzing the TH or TM treatments, we detect (among others) an enrichment of the GO-terms PERK-mediated unfolded protein response (GO:36499) and regulation of response to ER stress (GO:1900101) (Fig. 1b) (GO:36499: TM ∼44-fold, *P*-value: 2.17 × 10e−6; TH ∼18-fold, *P*-value: 1.1 × 10e−2; GO:1900101: TM ∼8-fold, *P*-value: 1.15 × 10e−2; TH ∼6-fold, *P*-value: 1.72 × 10e−4). When restricting the analysis to the transcripts that are co-regulated in both treatments, both GO-terms become even further enriched (more than 75-fold and 14-fold, respectively). Comparable results are obtained when gene expression changes after 6 h of treatment are analyzed.

Similar to transcriptional regulation, the analysis of ribosome-protected fragments indicates that treatment with TM or TH triggers largely different cellular responses (Fig. 1d, Supplementary Fig. 4D, E). Although after 2 h of treatment, the number of co-regulated genes is rather low (14 up, 41 down, Fig. 1d and Supplementary Fig. 3), a significant number of co-upregulated genes can be detected after 6 h. Among the 257 genes that exhibit a significant increase of ribosome-protected fragments, we again detect a strong enrichment of factors that are functionally connected to the UPR as reflected by the enrichment of UPR-associated GO-terms.

In sum, this validates our correlative multi-omics approach and demonstrates that stringent filtering allows to further enrich the UPR-driven changes.

### The UPR regulon

We find that adaptive changes to gene expression that are shared between treatment with TM or TH are strongly enriched for known UPR-mediated gene regulation. This suggests that other genes that exhibit a similar expression signature, also are likely under the control of the same pathway, the UPR. By analyzing changes to the transcriptome, the proteome, and to ribosome occupancy that are found in both treatments, we can now define the UPR regulon.

Here we focus on genes that encode proteins that show increased synthesis after treatment, suggesting a function in the ER stress response pathway (Supplementary Data 1). Genes that are induced on the level of the RNA (as judged by differential expression after treatment) can be considered to be part of the UPR regulon sensu stricto. However, if the increase in RNA abundance is not matched by increased protein synthesis or protein abundance (as determined by ribosome profiling or MS), it is unlikely that the encoded protein contributes to the ER stress response and therefore the respective locus is not considered. Similarly, an increase in protein abundance (as detected by MS), which is not paralleled by an increase in protein synthesis (as determined by ribosome profiling) most likely reflects altered protein turnover and therefore the respective genes are not considered to be directly regulated by the UPR.

Among the 267 genes that, according to these criteria, are induced by ER stress, we identify 37 factors that have previously been implicated in the UPR pathway. Moreover, we confirm the induction of numerous ATF4-responsive loci, such as genes that encode Vascular Endothelial Growth Factor A (VEGFA)^36^, Eukaryotic Translation Initiation Factor 4E Binding Protein 1 (EIF4EBP1)^37^, and several aminoacyl-tRNA synthetases (AARS, CARS, EPRS, GARS, MARS, SARS, and WARS)^38,39^. We also detect the induction of cancer-relevant genes that have been linked to stress response pathways other than the UPR (such as the cell cycle regulator and proto-oncogene Polo-like kinase 3, PLK3), or to inflammation (such as Nuclear Factor Kappa B Subunit 2, NFKB2, or Prostaglandin-Endoperoxide Synthase 2, PTGS2)^40–42^. The majority of proteins that we find induced by the UPR, however, have not been previously implicated in any stress response pathway.

### Experimental validation of UPR-driven induction of SLFN5

To validate that these loci are induced by the UPR, we selected one of the candidates that was upregulated after TM and TH treatment but for which no connection to a stress response pathway had been previously reported: Schlafen 5 (SLFN5). The encoded protein functions as a transcriptional regulator and has been linked to cancer progression, invasive growth, and patient survival^39,43–46^.

**Fig. 4:**
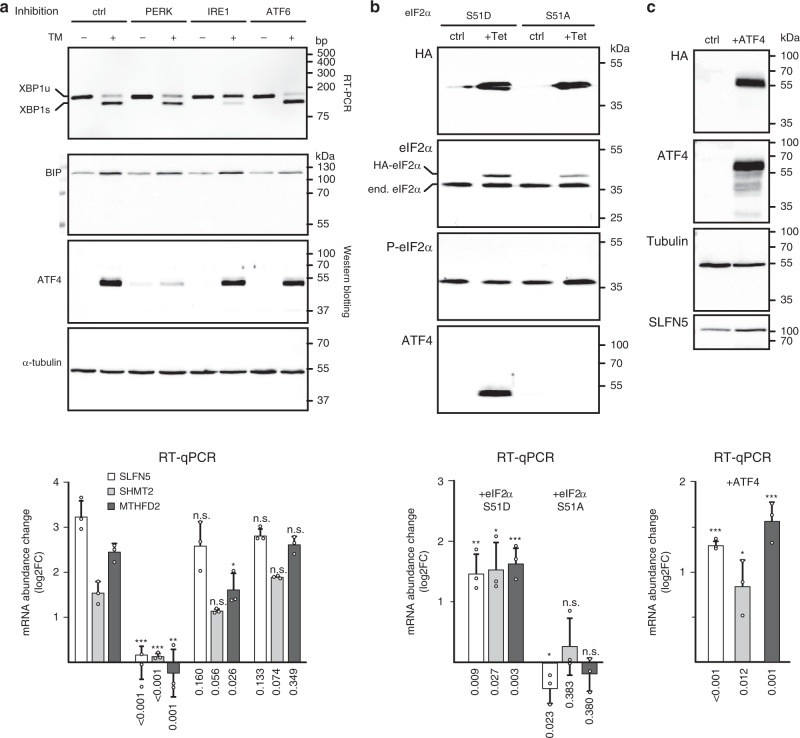
SLFN5, SHMT2, and MTHFD2 are induced by the UPR via the PERK–EIF2S1–ATF4 pathway. **a** Gene expression analysis of SLFN5, SHMT2, and MTHFD2 mRNAs after activation of the UPR in LN-308 cells. In parallel to stimulation with TM (lanes denoted +), either PERK, IRE1, or ATF6 were inhibited with small molecules (as indicated at the top). DMSO treatment served as a control. After 6 h, changes to gene expression were monitored. Top panel: RT-PCR analysis of IRE1-mediated cytoplasmic processing of XBP1 mRNA. Middle panels: Western blotting analyses of BiP (HSPA5), ATF4 and *α*-tubulin protein levels. Bottom: analysis of SLFN5 (white bars), SHMT2 (light gray bars) and MTHFD2 (dark gray bars) mRNA abundance by RT-qPCR. Plotted are mRNA abundance changes (log2 FC) relative to the control treatment and normalized to GAPDH mRNA levels. **b** Expression analysis of SLFN5, SHMT2, and MTHFD2 mRNA abundance in HEK293 cells after expression of a phosphomimetic eIF2a protein (lanes S51D), or a non-phosphorylatable variant (lanes S51A). After 24 h of induction (lanes + Tet), expression of the mutant proteins is monitored relative to control samples (ctrl). Top panels: Western blotting analyses with antibodies that recognize either the HA-tag of the stably transfected eIF2a protein-encoding constructs (top panel), total eIF2*α* (second panel), S51 phosphorylated eIF2*α* (third panel), or ATF4. Bottom: analyses of mRNA abundance as described for **a**. **c** Expression analysis of SLFN5, SHMT2, and MTHFD2 mRNA abundance in LN-308 cells after transient expression of ATF4. Western blotting analyses of protein expression using antibodies specific for the HA-tag of the transfected construct, ATF4, tubulin, or SLFN5. Bottom: RT-qPCR analysis as described for **a**. Experiments were performed in at least three biologically independent experiments, representative blots/gels are shown (uncropped images are provided in the supplementary information). Molecular weight markers (in kDa) or DNA size markers are indicated on the right of each panel. qPCR data are represented as mean ± SD of three biologically independent experiments measured in technical triplicates (dots represent average values of three technical replicates). *P*-values are provided below each bar, n.s. not significant *P* > 0.05; *P < 0.05, **P < 0.05, ***P < 0.01 as determined by a Student’s *t*-test (two-sided, true variance equality, confidence level 0.95, no adjustment for multiple comparisons).

To confirm that the induction of SLFN5 is driven by the UPR, we analyzed mRNA expression after treatment with TM while simultaneously inhibiting individual branches of the UPR by pharmacological treatment (Fig. 4a). Efficiency of the treatments was confirmed by probing for IRE1-mediated processing of XBP1, PERK-mediated induction of ATF4, and expression of HSPA5, which is sensitive to inhibition of ATF6. Inhibition of IRE1 (with STF-083010) or ATF6 (with CeapinA7)^47^ had no measurable effect on TM-induced accumulation of SLFN5 mRNA in LN-308 and HEK293 cells, while PERK inhibition (with GSK2606414) completely abolished the effect. Furthermore, induction of SLNF5 mRNA expression and protein accumulation could be recapitulated by the expression of a phosphomimetic mutant of EIF2S1 (S51D) in HEK293 cells but not by expression of the non-phosphorylatable mutant EIF2S1-S51A (Fig. 4b). Similarly, transient expression of ATF4 in LN-308 cells (but not of a control protein) results in accumulation of SLFN5 mRNA (Fig. 4c). This experimentally confirms induction of SLFN5 by the UPR through the PERK–EIF2–ATF4 pathway.

### Metabolic rewiring by induction of the UPR

**Fig. 5:**
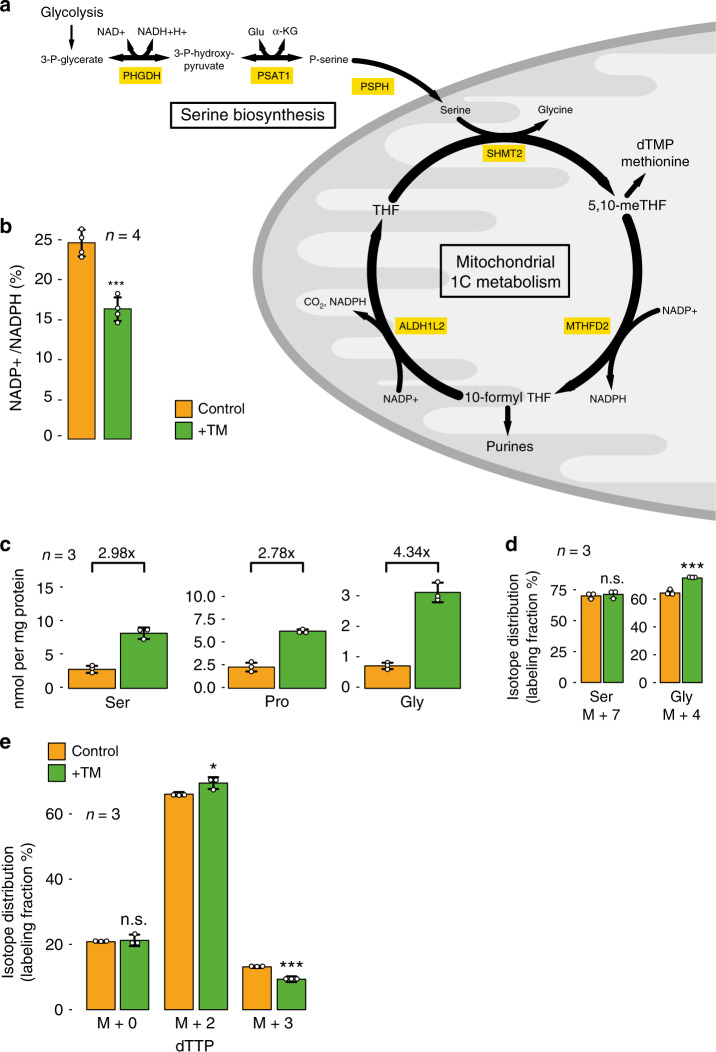
The UPR induces rewiring of cellular metabolism. **a** Schematic depiction of the UPR-induced enzymatic pathway that diverts intermediary metabolites from glycolysis to fuel mitochondrial one-carbon metabolism via serine biosynthesis. PHGDH: phosphoglycerate dehydrogenase, PSAT1: phosphoserine aminotransferase 1, PSPH: phosphoserine phosphatase, SHMT2: serine hydroxymethyltransferase 2, MTHFD2: bifunctional methylenetetrahydrofolate dehydrogenase/cyclohydrolase, ALDH1L2: aldehyde dehydrogenase 1 family member L2, THF: tetrahydrofolate, 5,10-meTHF: 5,10-methylene THF. **b** Analysis of the redox status of the nicotinamide adenine dinucleotide phosphate pool in LN-308 cells after 24 h of control treatment with DMSO (yellow bar), or treatment with TM (green bar). *P*-value: 0.0003. **c** Analysis of the cellular concentration of the amino acids serine, proline, and glycine after induction of the UPR (green bars) relative to control treatment with DMSO (yellow bars). Fold-increase values after 24 h of treatment are displayed at the top of each panel. **d** Analysis of the isotope distribution of serine and glycine 24 h after application of a metabolically labeled serine tracer. Cells were either control treated with DMSO (yellow bars) or stimulated for 24 h with TM (green bars). *P*-values: Serine M + 7: 0.590, Glycine M + 4: 0.001. **e** Analysis of the contribution of mitochondrial folate metabolism to thymidylate synthesis in control-treated LN-308 cells (yellow bars), or after induction of the UPR with TM (green bars). The isotope distribution of dTTP was analyzed 24 h after administration of a metabolically labeled serine tracer. *P*-values: dTTP M + 0: 0.713, dTTP M + 2: 0.038, and dTTP M + 3: 0.002. All data are represented as mean ± SD of three (**c**–**e**) or four (**b**) biologically independent experiments. A Student’s *t*-test was used for the statistical testing with the following parameters: two-sided, true variance equality and confidence level at 0.95. No adjustment for multiple comparisons was made. n.s. not significant *P* > 0.05, *P < 0.05, ***P < 0.005. Source data are provided as a Source Data file.

In particular under stress conditions, proliferating cells need to finely coordinate their gene expression, biosynthetic, and bioenergetic programs to cope with the stress and to allow for anabolic growth and proliferation. Metabolic reprogramming is a hallmark of cancer, which e.g. results in an increased conversion of glucose into lactate (which is known as the Warburg effect). In our data, we detect upon induction of the UPR an increased expression of enzymes involved in a metabolic pathway that channels intermediary metabolites from glycolysis to the folate-mediated one-carbon (1C) metabolism via serine biosynthesis (Fig. 5a). 1C metabolism has an important role in amino acid homeostasis, maintenance of epigenetic modifications and the oxidative state of the cell, and the production of nucleobases (purines and thymidylate) that are required for cellular proliferation^48,49^. Therefore, it is not surprising that enzymes of the 1C metabolism, in particular bifunctional methylenetetrahydrofolate dehydrogenase/cyclohydrolase (MTHFD2), were identified to be among the most differentially expressed enzymes in cancer^50,51^. Moreover, the overexpression of MTHFD2 is associated with proliferation of tumor cells^52^ and poor prognosis in breast cancer patients^50,53^. Similarly, an increase in serine biosynthetic capacity contributes to oncogenesis in various tumors and it has been suggested that phosphoglycerate dehydrogenase (PHGDH), which catalyzes the first step in serine biosynthesis, is a potential oncogene^54–56^.

Upon treatment with either TM or TH, we detect upregulation of the three enzymes that catalyze conversion of 3-phosphoglycerate into serine (via 3-phospho-hydroxypyruvate and phosphoserine): phosphoglycerate dehydrogenase (PHGDH), phosphoserine aminotransferase 1 (PSAT1), and phosphoserine phosphatase (PSPH). In addition, we observe the induction of proteins involved in mitochondrial 1C metabolism: serine hydroxymethyltransferase 2 (SHMT2), bifunctional methylenetetrahydrofolate dehydrogenase/cyclohydrolase (MTHFD2), and aldehyde dehydrogenase 1 family member L2 (ALDH1L2). We also confirmed that SHMT2 and MTHFD2 expression is induced in a UPR-dependent fashion by demonstrating that the accumulation of mRNAs encoding these proteins is sensitive to PERK inhibition and can be mimicked by expression of either a phosphomimetic version of EIF2S1, or the transcription factor ATF4 (Fig. 4).

Previously, it has been demonstrated that the expression of all three enzymes involved in serine biosynthesis (PHGDH, PSAT1, and SHMT2) and select enzymes of mitochondrial 1C metabolism can be induced by stress response pathways and/or increased expression of ATF4^39,56,57^. However, induction of the entire metabolic pathway involved in mitochondrial turnover of folates has not been reported so far and the possible implications e.g. for chemotherapeutic treatment have not been assessed.

To indirectly confirm that induction of the UPR increases the expression of the aforementioned metabolic enzymes, we analyzed cell growth in conditions where proliferation is limited by the cells’ ability to synthesize serine. Withdrawal of the non-essential amino acid serine from the medium impaired cell growth by ~21%; however, after administration of TM, the effect was less pronounced (~12% relative to the control) supporting the hypothesis that induction of the UPR can increase the serine biosynthetic capacity, partially alleviating growth impairment in serine-depleted medium (Supplementary Fig. 8).

To understand whether the UPR also increases the metabolic flux through the mitochondrial 1C metabolism, we analyzed the redox state of the NADP pool. After induction of the UPR, we found a significant decrease in the NADP/NADPH ratio (Fig. 5b). As NADPH can be generated via several different metabolic pathways including 1C metabolism, we performed a metabolic labeling experiment to further assess flux of metabolites through the 1C metabolism adapted from^58^. We cultured LN-308 cells in the presence of a stable isotope-labeled [1,2,3-^13^C; 2,3,3-^2^H, 2-^15^N]serine tracer and analyzed abundance and labeling efficiency of serine, glycine, proline, and dTTP 24 h after induction of the UPR relative to non-stressed cells (Fig. 5c–e and Supplementary Fig. 6). As expected, due to the attenuation of translation, we observed an increase in the free amino acids serine, glycine and proline after induction of the UPR. Although serine and proline levels were increased 2.98-fold and 2.78-fold, respectively, the increase in glycine was much more pronounced (4.34-fold, Fig. 5c). Moreover, while the fraction of labeled serine was unaffected by the UPR, we could detect a significant increase in labeled glycine (M + 4) produced from labeled serine (M + 7) by serine hydroxymethyltransferase activity (Fig. 5d). This suggests an increased flux of metabolites through 1C metabolism in LN-308 cells after TM treatment.

To assess whether cytosolic or mitochondrial 1C metabolism is increased, we analyzed the isotope distribution of dTTP after labeling with the heavy serine tracer. Cytosolic production of 5,10-methylene-THF results in dTTP production with an increased mass of +3, whereas a mass increases of only +2 Dalton is indicative of metabolization of serine in mitochondria (Supplementary Fig. 6)^58^. After induction of the UPR, we observe a significant increase in M + 2 dTTP and concomitantly a decrease in dTTP M + 3, whereas the background of non-labeled dTTP remains unchanged (Fig. 5e). This confirms an increased activity of mitochondrial over cytosolic 1C metabolism.

### EIF2S1 phosphorylation induces chemoresistance

**Fig. 6:**
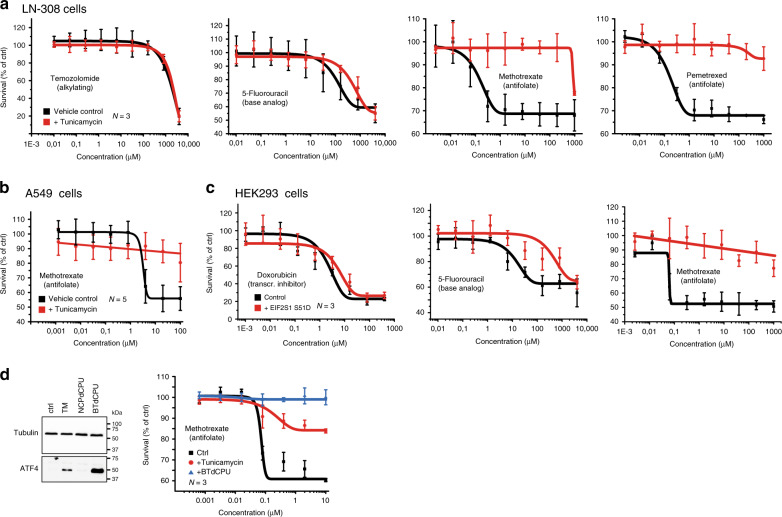
Stress-mediated phosphorylation of EIF2S1 induces resistance to the folate-based antimetabolites methotrexate and pemetrexed. Survival of LN-308 cells (**a**) and A459 cells (**b**) after 24 h of treatment with the indicated concentrations of the FDA-approved chemotherapeutic reagents in the absence (black lines, vehicle control) or presence of tunicamycin (red lines). **c** Survival of stably transfected HEK293 cells before (black lines, vehicle control) and after induction of expression of a phosphomimetic mutant of EIF2S1 (S51D) (red lines). Cells were treated for 24 h with the indicated concentrations of FDA-approved chemotherapeutic reagents before scoring survival. **d** Survival of LN-308 cells in the presence of MTX after activation of the kinase HRI. Left panel: representative western blots of ATF4 and beta-tubulin protein levels after stimulation with the HRI activator BTdCPU, or the control compound NCPdCPU. Treatment with DMSO or TM served as control. Right panel: Cell survival in the presence of the indicated concentrations of MTX of control-treated LN-308 cells (black), TM-treated (red), or BTdCPU-treated LN-308 cells (blue). Data are represented as mean ± SD of three (**a**, **c**, and **d**) or five (**b**) biologically independent experiments. Representative western blots of at least three biologically independent replicates are depicted; pictures of the uncropped membranes are provided in the supplementary information.

Folate metabolism is the target of antimetabolite drugs that are widely applied for the treatment of inflammatory diseases and malignancies. To assess whether metabolic rewiring and stimulation of mitochondrial 1C metabolism changes the response to treatment with folate-based antimetabolites, we determined upon induction of the UPR the dose–response curves to several FDA-approved drugs with different modes of action. Sensitivity to treatment with the intercalating reagent Doxorubicin and the alkylating compound temozolomide (TMZ) is unchanged after induction of the UPR (Fig. 6a). Similarly, the half maximal inhibitory concentration (IC_50_) of 5-fluoro-uracil, a suicide inhibitor of thymidylate synthase (TYMS), remains unaffected. In contrast, after the induction of the UPR, the cells become resistant to treatment with the folate derivatives methotrexate (MTX) and pemetrexed as evidenced by an ~10,000-fold increase in IC_50_ after stimulation with TM (Fig. 6a). This is not unique to LN-308 cells, as resistance to treatment with MTX after induction of the UPR can also be observed in the adenocarcinoma-derived cell line A549 (Fig. 6b). Importantly, after expression of a phosphomimetic EIF2S1 (S51D) mutant protein, resistance to treatment with MTX can also be observed in HEK293 cells, which in contrast to the malignantly transformed LN-308 and A549 cell lines are not of cancerous origin but have been immortalized by transfection of adenovirus DNA (Fig. 6c).

The central role of EIF2S1 phosphorylation in the UPR-mediated induction of serine biosynthesis, mitochondrial 1C metabolism, and resistance to MTX suggests that other stress kinases that phosphorylate EIF2S1 could similarly induce resistance to folate-based antimetabolite therapy. This includes, besides PERK, the kinases general control nonderepressible 2 (GCN2, activated by e.g. amino acid deprivation), protein kinase R (PKR, activated by double-stranded RNA e.g. upon viral infection), and heme-regulated inhibitor (HRI, activated upon oxidative stress, heme deficiency, osmotic shock and heat shock). Together these kinases constitute a common adaptive stress-signaling pathway, the integrated stress response (ISR). Signaling during activation of the ISR is, however, complex and can result in different cellular responses and cell fate. Moreover, the major transcription factor downstream of EIF2S1, ATF4, acts in combination with other proteins that influence its activity and the cellular outcome of ISR signaling^59^, among them DDIT3, ATF3, and TRIB3 that we detect to be upregulated upon induction of the UPR. We therefore assessed whether MTX resistance could also be induced upon activation of another ISR kinase apart from PERK. For this, we treated LN-308 cells with a small molecule activator of HRI (BTdCPU) or a chemically related control compound (NCPdCPU)^60^. As expected, accumulation of ATF4 protein could only be observed after stimulation with BTdCPU, but not after treatment with the control compound. Concomitantly, we again detected resistance to treatment with MTX (Fig. 6d). Moreover, treatment with Salubrinal, an inhibitor of EIF2S1 phosphatases that impairs eIF2 function independent of the upstream kinases such as PERK, induces partial resistance to MTX in LN-308 cells, supporting the notion that eIF2S1 phosphorylation is a key event to trigger resistance (Supplementary Fig. 7).

Taken together, this demonstrates that stress-mediated induction of chemoresistance to folate-based antimetabolites is (a) driven by phosphorylation of EIF2S1 and (b) appears to be a resistance mechanism that can be broadly activated in various different cell types irrespective of malignant transformation.

Resistance to MTX treatment has previously been attributed mainly to (a) mutation or increased expression of its target protein dihydrofolate reductase (DHFR), (b) a change in the ratio of MTX and its polyglutamated form (MTX-PG) mediated by reduced activity of folylpolyglutamate synthase (FPGS) or an increase in gamma-glutamyl hydrolase (GGH) activity, or (c) reduced cellular uptake of MTX or increased efflux mediated by transporter proteins for xenobiotics^61^. Several proteins have been implicated in the transport of MTX, including the solute carrier family proteins SLC19A1 (aka reduced folate carrier, RFC) and SLC46A1 (aka proton-coupled folate transporter, PCFT) and the ATP-binding cassette transporters ABCC1-5 (aka multidrug resistance-associated proteins 1–5, MRP1-5), ABCG2 (aka breast cancer resistance protein, BCRP), and ABCB1 (aka multidrug resistance protein 1, MDR1)^62–64^. Upon induction of the UPR, we do not find any of the aforementioned proteins (or related transporters) to be significantly regulated on the level of translation or protein abundance (we only detect a small but significant decrease in RPF abundance for ABCC4 after 2 h but not 6 h of TM treatment and for ABCC5 and SLC46A1 after 6 h of TH treatment, see Supplementary Data 3). Also, the levels of DHFR, FPGS and GGH remain unchanged suggesting that resistance to MTX is driven by a different and previously unrecognized pathway.

TYMS is the key enzyme in the sole pathway for de novo thymidylate production in higher organisms, which is required for DNA replication and cellular proliferation. Its activity is closely connected to folate metabolism: to synthesize deoxythymidine monophosphate (dTMP), TYMS catalyzes reductive methylation of deoxyuridine monophosphate (dUMP), using a carbon group from 5,10-methylene tetrahydrofolate (5,10-meTHF) and converting it to dihydrofolate (DHF). Reduction of DHF by DHFR generates tetrahydrofolate (THF), which can then again participate in the folate cycle and accept another carbon group. MTX and pemetrexed inhibit DHFR and block reduction of DHF thus depleting THF. It remains to be tested whether the UPR-mediated increase in SHMT2, MTHFD2, and ALDH1L2 allows sustaining of the folate cycle even under conditions when THF concentrations are decreased by inhibition of DHFR, resulting in resistance to treatment with folate-based antimetabolites.

## Methods

### Cloning

The pcDNA5/FRT/TO vector (ThermoFisher) was modified to contain a 3HA-tag and additional restriction sites, generating pcDNA5/FRT/TO-HA. The EIF2S1 (NM_004094, [https://www.ncbi.nlm.nih.gov/nuccore/NM_004094.5]) and ATF4 (NM_001675, [https://www.ncbi.nlm.nih.gov/nuccore/NM_001675]) open reading frames were amplified by RT-PCR from total RNA extracted from LN-308 cells and, after sequence verification, cloned into the pcDNA5/FRT/TO-HA vector, generating pcDNA5/FRT/TO-HA-EIF2S1 and –ATF4. PCR-mediated site-directed mutagenesis was performed to introduce mutations in EIF2S1 that replace the Ser51 codon (TCC) by codons that either encode aspartic acid (GAC) or alanine (GCC), generating pcDNA5/FRT/TO-HA-EIF2S1-S51D and –S51A.

### Cell culture

Flp-In^TM^ T-REx^TM^ HEK293 (ThermoFisher, RRID:CVCL_U427) and LN-308 (RRID:CVCL_0394) cells were cultured in DMEM supplemented with 10% FBS, 100 U/ml penicillin and 100 µg/ml streptomycin at 37 °C and at 5% CO_2_. To induce ER stress, the medium was supplemented with 200 nM Thapsigargin or 2.5 µg/ml Tunicamycin (dissolved in DMSO); DMSO only served as a control. To inhibit individual branches of the UPR after stimulation with TM, cells were treated additionally with either 50 µM STF-083010, 1 µM GSK2606414, or 1 µM CeapinA7. For the activation of HRI, BTdCPU (activator) and NCPdCPU (control compound) were used at concentrations of 10 µM. Salubrinal was used at a concentration of 50 µM to inhibit dephosphorylation of EIF2S1.

Stable Flp-In T-Rex HEK293 cell lines were generated according to the manufacturer’s instructions (ThermoFisher) using pcDNA5/FRT/TO-HA-EIF2S1-S51D or –S51A plasmids that encode HA-tagged phosphomimetic (S51D) or non-phosphorylatable (S51A) eIF2*α* mutant proteins under the control of an inducible promoter. For the transfection lipofectamine 2000 (ThermoFisher) was used according to the manufacturer’s instructions, followed by selection of positive clones in the presence of 10 µg/ml Blasticidin and 100 µg/ml Hygromycin B. After induction with 1 µg/ml tetracycline for 24 h, expression of the mutant proteins was confirmed by immunoblotting.

For the transient expression of ATF4, LN-308 cells were transfected with a pcDNA5/FRT/TO-HA-ATF4 using lipofectamine 2000 according to the manufacturer’s instructions.

### Immunoblotting

Cells were lysed for 20 min on ice in RIPA buffer (150 mM NaCl, 50 mM Tris pH 8.0, 1% NP-40, 0.5% sodium deoxycholate, 0.1% SDS, and 1× cOmplete protease inhibitor cocktail (Roche)) followed by clearing of the lysates by centrifugation at 4 °C for 10 min at 17,000×*g*. Equal amounts of total protein were denatured for 5 min at 95 °C in sample buffer (50 mM Tris pH 6.8, 1%SDS, 10% glycerol, 100 mM DTT, 0.1% bromophenol blue), separated by SDS-PAGE and transferred onto nitrocellulose membranes (Amersham Protan 0.2 µm NC). Blocking occurred in TBS-T (10 mM Tris pH 7.5, 150 mM NaCl, 0.1% Tween 20) supplemented with 3% (v/v) BSA or TBS (10 mM TRIS, 150 mM NaCl) supplemented with 5 % (v/v) non-fat dry milk powder. The following primary antibodies were used: anti-BIP (Cell Signaling Technology 3177, RRID:AB_2119845, 1:1000), anti-IRE1 (Abcam ab96481, RRID:AB_10679929, 1:1000), anti-XBP1 (Biolegend 619501, RRID:AB_315907, 1:500), anti-ATF4 (Cell Signaling Technology 11815, RRID:AB_2616025, 1:1000), anti-eIF2a (Cell Signaling Technology 5324, RRID:AB_10692650, 1:1000), anti-eIF2α-pS51 (Abcam ab32157, RRID:AB_732117, 1:2000), anti-alpha-tubulin (Sigma DM1A, RRID:AB_477593, 1:4000), anti-beta-actin (Sigma A2066, RRID:AB_476693, 1:100), anti-PERK (Cell Signaling Technology 3192, RRID:AB_2095847, 1:1000), anti-SLFN5 (Sigma HPA017760, RRID:AB_2189993, 1:1000), and anti-HA (Sigma HA-7, RRID:AB_262051, 1:1000). For detection either horseradish-peroxidase-coupled antibodies (Peroxidase AffiniPure Goat Anti-Mouse IgG, Light Chain Specific, or Peroxidase IgG Fraction Monoclonal Mouse Anti-Rabbit IgG, Light Chain Specific, Jackson ImmunoResearch, both 1:10,000) or IRDye-coupled antibodies (Licor #925-32211, RRID:AB_2651127, #925-68071, RRID:AB_2721181, and #925-68020, RRID:AB_2687826, all 1:5000) were employed. Detection occurred either by ECL (Clarity Western ECL Substrate and ChemiDoc Imaging System, BioRad), or with the Odyssey CLx imaging system (Licor).

### RT-PCR analyses

Total cellular RNA was extracted using TRIzol (ThermoFisher) reagent according to the manufacturer’s instructions. 2.5 µg of RNA were reverse transcribed in a 20 µl reaction using an oligo-dT primer and SuperScript III (ThermoFisher) for 1 h at 50 °C. To analyze XBP1 splicing, 25 cycles of PCR were performed using Phusion High-Fidelity DNA Polymerase (NEB). For quantitative real-time PCR, per reaction 1 µl of a 1:5 dilution of the RT reaction, 250 nM primers and SsoFast EvaGreen Supermix (BioRad) were used and analyzed using a CFX96 Real-Time PCR Detection System (BioRad). All primer-sets were tested for amplification efficiency and generation of a single product (by melting curve analysis and gel electrophoresis). Changes to expression levels were calculated relative to a control gene (GAPDH) according to the mathematical model by Pfaffl^65^. Sequences of all primers and additional information are provided in Supplementary Data 5.

### RNA-sequencing library preparation and sequencing

Total RNA was extracted using TRIzol reagent (ThermoFisher), RNA integrity was checked using the RNA Nano 6000 Assay Kit of the Bioanalyzer 2100 system (Agilent Technologies, Santa Clara, CA), and concentration was measured with Qubit® RNA Assay Kit in Qubit® 2.0 Fluorometer (ThermoFisher). Starting with ∼300 ng of total RNA as input, ribosomal RNA was removed by NEBNext rRNA Depletion Kit (Human/Mouse/Rat) (New England Biolabs). Subsequently, stranded total RNA-seq libraries were prepared using the NEBNext® Ultra™ II Directional RNA Library Prep Kit for Illumina® (New England Biolabs) according to the manufacturer’s instructions. RNA-seq barcoded libraries that passed the QC step, which was assessed on the Agilent Bioanalyzer system, were then pooled in equimolar amounts; 1.8pM solution of this pool were loaded on the Illumina sequencer NextSeq 500 and sequenced bi-directionally (80 nt read length).

### Ribosome profiling

TH- or TM-stimulated cells were treated with 0.1 mg/ml cycloheximide for 5 min. After washing with ice-cold PBS (supplemented with cycloheximide), cells were harvested and lysed for 15 min on ice in a buffer containing 5 mM Tris pH 7.4, 1.5 mM KCl, 5 mM MgCl_2_, 0.5% Triton-X, 0.5% sodium deoxycholate, 0.1 mg/ml cycloheximide, RNasin and 1× cOmplete protease inhibitor cocktail (Roche). The cell lysate was cleared by centrifugation and digested with 0.2 U RNaseI (Ambion) per 1 µg RNA for 15 min at 4 °C. To stop the reaction, 1 U SUPERaseIn RNase Inhibitor (ThermoFisher) was added per unit of RNaseI.

Ribosomal complexes were separated on 12 ml 10–50% sucrose gradients (in a buffer containing 20 mM Tris pH 7.4, 75 mM NaCl, 5 mM MgCl_2_, 0.1 mg/ml cycloheximide, and 1 mM DTT) by centrifugation at 4 °C and 35,000 rpm for 3 h in a SW41 rotor. Fractions were collected at 0.5 ml/min with continuous monitoring of conductivity and UV absorption at 254 nm. Fractions containing 80S monosomes were diluted with an equal amount of RNase-free water followed by organic extraction with Phenol:Chloroform:Isoamylalkohol 25:24:1 (Roth) and ethanol precipitation. RNAs were then separated by denaturing Urea-PAGE and visualized by SYBR gold staining. RNAs with a length of 27–33 nt were excised from the gel and eluted overnight in a buffer containing 300 mM NaOAc pH 5.5, 1 mM EDTA, and 0.25% SDS followed by ethanol precipitation.

After dephosphorylation for 1 h at 37 °C with 20 U of T4 Polynucleotide Kinase (New England Biolabs), RNAs were ligated to a universal miRNA cloning linker (New England Biolabs) using truncated T4 RNA ligase 2. After gelpurification reverse transcription was performed using SuperscriptIII reverse transcriptase (ThermoFisher) and a primer complementary to the ligated adapter, followed by alkaline hydrolysis of the RNA strand. The resulting cDNAs were gelpurified and contaminating sequences originating from rRNA were depleted by substractive hybridization to a mixture of biotinylated DNA oligonucleotides (for sequences see Supplementary Data 5) and Streptavidin magnetic beads (New England Biolabs). The recovered RNAs were then circularized with CircLigase II ssDNA Ligase (Epicenter) according to the manufacturer’s instructions and subjected to PCR amplification to generate amplicons suitable for Illumina sequencing. After gelpurification, DNA concentration was determined using a Qubit Fluorometer (Qubit 2.0, ThermoFisher Scientific). Bioanalyzer or TapeStation (Agilent) analyses were performed to assess the quality of the samples followed by deep sequencing on an Illumina HiSeq platform.

### Quantification and statistical analysis of transcriptome

All statistical data analysis was performed in R^66^ and Python^67^. Demultiplexed sequences were subjected to adapter trimmed using trimmomatic^68^ Version 0.38 and aligned to GENCODE^69^ reference annotations for the human genome (GRCh38.primary_assembly, gencode.v26.annotation) using STAR^70^ Version 2.5.4b. Gene level expression were quantified using HTSeq^71^ and raw RNA-Sequencing count data normalized and fold changes computed using the DESeq2 library^72^ with the default DESeq function that estimated size factors and dispersion, performed negative binomial GLM fitting and applied Wald statistics for the test of significance of the comparison treated vs. untreated. Experiments were performed in biological replicates, except for the treatment with TM where technical replicates were analyzed (experimental numbers are summarized in the supplemental information).

Ribosome loading scores were computed from ribosomal profiling and RNA-sequencing data using RiboDiff^73^. For comparison, log2 transformation was used for all experimental datasets. The cut-off for differential expression was set at false-discovery rate of 5%. Differential expression of RNA-Seq and ribosomal profiling datasets was determined based 95% confidence interval. For RNA-Seq and ribosomal profiling datasets, only protein-coding regions were considered in further analysis.

### Proteomics sample preparation

Cells were pre-treated with TM or TG as described above and lysed in 50–200 µl lysis buffer containing 1% SDS, 150 mM NaCl, 50 mM Tris-HCl (pH 7.8), 1× cOmplete™ (Roche). For each treatment and each incubation time, three biological replicates were performed. After lysis, 1 µl Benzonase® Nuclease (Merck KGaA) per 300 µl and an equal volume of 1 M MgCl2 were added to each sample followed by incubation for 30 min at 37 °C. The cell debris was clarified by centrifugation at 18,000 × *g* for 30 min and at 4 °C. The total protein amount was determined using bicinchoninic acid assay (Pierce™ BCA Protein Assay Kit, ThermoFisher Scientific™). The disulfide bonds were reduced with 10 mM Tris(2-carboxyethyl)phosphine hydrochloride (TCEP, Sigma Aldrich) at 56 °C for 30 min and free sulfhydryl groups were alkylated with 30 mM Iodoacetamide (IAA, Sigma Aldrich) for 30 min at 25 °C in the dark. The proteolytic digestion followed a filter-aided sample preparation protocol (FASP) using a centrifugal device (PALL Nanosep, 30 kDa molecular weight cut-off). The SDS concentration in cell lysate was reduced to <0.2% by addition of Urea buffer (8 M Urea in 100 mM Tris-HCl pH 8.5) before loading onto the centrifugal device. All the centrifugation steps were performed at 13,500 × *g* at 25 °C. The membrane of the centrifugal device was equilibrated with 100 µl Urea buffer, followed by loading the sample and subsequently washing three times with 100 µl 50 mM NH4HCO3 (pH 7.8). The enzymatic digestion occurred at 37 °C for a maximum of 15 h using 100 µl digestion buffer containing Trypsin (Trypsin Gold, Promega) at a ratio of 1:25 (w/w, protease to substrate), 0.2 M Guanidine-HCl, 2 mM CaCl_2_ and 50 mM NH_4_HCO_3_ (pH 7.8). The tryptic peptides were collected by adding 100 µl 25 mM NH4HCO3 (pH 7.8) and the digestion was stopped by acidifying with 10 µl 10% trifluoroacetic acid (TFA). The quality of the samples was examined using a monolithic reverse phase separation method according to Burkhart et al.^74^. For further analysis, the peptide solution was stored at −80 °C.

### LC–MS/MS analysis

For the separation of peptides, an Ultimate 3000 Rapid Separation Liquid Chromatography (RSLC, Thermo Scientific) nanoLC system was used. 1 µg of peptides was loaded for each measurement. The peptides were first pre-concentrated on a pre-column (Acclaim C18 PepMap100, 100 µm 2 cm, ThermoFisher Scientific) using 0.1% TFA at a flowrate of 20 µl/min and then loaded on an analytical main-column (Acclaim C18 PepMap100, 75 µm 50 cm, Thermo Scientific). A linear gradient employing A: 0.1% formic acid (FA) and B: 84% acetonitrile (ACN), 0.1% FA running from 3 to 35% B in 180 min at a flowrate of 250 nl/min was used. For the global proteomics analysis, the high resolution orbitrap mass spectrometer Q Exactive HF (Thermo Scientific) was used in top 15 data-dependent acquisition mode. Full MS scans were acquired at a resolution of 60,000 full width at half maximum (FWHM), AGC target: 1e6 and maximum injection time of 120 ms. Data-dependent MS/MS scans were acquired on the 15 most abundant ions using a normalized collision energy of 27%, at a resolution of 15,000 FWHM, AGC target: 5e4, isolation width: 1.6 *m*/*z*, fixed first mass: 120 *m*/*z* and dynamic exclusion of 20 s. MS/MS scans were performed only for precursor ions with charge states between 2 and 4.

### Proteomics data analysis

For the data analysis of mass spectrometry data, Progenesis QI for Proteomics software (version 3.0 NonLinear Dynamics) was used. X!Tandem^75^ via SearchGUI interface version 2.5.0^76^ and Mascot 2.4 (Matrix Science) were used as search algorithm for the peptide identification. For the data base search, Uniprot human database (downloaded on 22nd of July 2015) was used with the following search parameters: fixed modification: carbamidomethylation at cysteine, variable modification: oxidation at methionine, trypsin as protease (maximum 2 missed cleavages), 10 ppm as MS1 tolerance and 0.02 Da as MS2 tolerance. PeptideShaker version 1.4.0^77^ was used to combine the peptide identification from X!Tandem and Mascot. Only proteins identified with at least 2 unique peptides were used for further analysis. The statistical data analysis was performed using R version 3.3.1 (codename “Bug in Your Hair”) using the *t*.test function (Student’s *t*-test, two-sided, true variance equality, confidence level at 0.95). The regulation cut-off was defined as median of the log2-fold-change ± twice the standard deviation of the log2-fold-change. The significant cut-off was defined as <0.05. The general calculation, data formatting and illustration of the data was performed using the R-packages reshape2, dplyr, ggplot2, and gridExtra.

### Functional annotation and multi-omics integration

All omics datasets were integrated via gene name—Uniprot protein id mapping. The datasets were integrated on the biological pathway level by performing GeneOntology—Biological process analysis on DAVID. All measured data points using corresponding experimental measurement platform were used as background for pathway analysis. All data transformations, UPR-related annotations and visualizations were performed in R 3.4.3.

### Analysis of cellular NADPH/NADP^+^ levels

LN-308 cells were treated for 24 h with 2.5 nM Tunicamycin (or DMSO as a control) and subsequently washed with PBS prior to harvesting. For each assay, 4 × 10^4^ cells were resuspended in 50 µl of PBS and lysed with an equal volume of a solution containing 1% DTAB in 0.2 N NaOH. NADPH and NADP^+^ concentrations were determined with the NADP/NADPH-Glo^TM^ assay (Promega) according to the manufacturer’s instructions. In brief, samples were split into aliquots of 50 µl each and subjected to treated either with acid (25 μl of 0.4 N HCl to determine NADP^+^ levels) or base (to determine NADPH levels). Samples were incubated for 15 min at 60 °C, cooled to room temperature and neutralized with Tris. 50 µl of each sample were then mixed with an equal volume of NADP/NADPH-Glo^TM^ detection reagent and after incubation for 45 min subjected to luminescence measurement using a Centro XS3 luminometer (Berthold).

### Labeling with serine and metabolite analyses

Metabolic labeling with a stable isotope-labeled serine was performed in a setup adapted from Ducker et al.^58^. In brief, LN-308 cells were first cultured in MEM (ThermoFisher) supplemented with 10% dialyzed FBS (Sigma Aldrich), 200 µM l-glutamine, and 400 µM l-serine for several passages. The cells were then washed twice with PBS before addition of medium in which the serine had been replaced by 400 µM of stable isotope-labeled, heavy serine (HO^13^C^2^H_2_-^13^C^2^H^15^NH_2_-^13^COOH, Cambridge Isotope Laboratories, Supplementary Fig. 6). After 24 h the cells were washed twice with PBS, harvested, snap frozen in liquid nitrogen and stored at −80 °C until further use.

Prior to the extraction of metabolites, each cell pellet was supplemented with 4 µl of an internal standard mixture containing 2.5 mM Serine-m + 3, 2.5 mM Glycine-m + 2, 2.5 mM Proline-m + 7 (all from Cambridge Isotope Laboratories) and 0.025 mM dTTP-m + 12 (Sigma Aldrich). 1 ml of cold 25% MeOH solution was added to each pellet. Cells were disrupted by three rounds of incubation in liquid nitrogen for 1 min followed by sonication in a water bath at 4 °C for 10 min. Proteins were precipitated for 1 h at −80 °C and centrifugation at 18,000×*g* for 30 min at 4 °C. Equal parts of the supernatant were dried under nitrogen flow before reconstitution in either 120 µl of 90% ACN (for HILIC-ESI-MS^2^ analysis) or in 120 µl of water for the measurement with IC-MS analysis.

The HILIC-ESI-MS analyses were performed on an Ultimate 3000 system coupled to a Q Exactive HF mass spectrometer (both from Thermo Scientific). The separation of Serine, Glycine and Proline was carried out on a Zic® HILIC column (150 × 1 mm, 3.5-µm particle size, 100 Å pore size, from Merck Darmstadt). The mobile phases were 90% acetonitrile (A) and 20 mM ammonium acetate in H_2_O at pH = 7.5 (B). The gradient eluted isocratically with 90% ACN for 2.5 min followed by an increase to 60% over 14 min and held at 60% for 2 min. Subsequent reconstitution of the starting conditions and re-equilibration with 100% A for 10 min resulted in a total analysis time of 35 min. 4 µl of sample were injected onto the column and the LC separation was carried out at 25 °C with a flow rate of 100 µl/min. The Q Exactive HF was equipped with a heated electrospray ionization source (HESI-II, Thermo Scientific) and was operated in positive ion mode. The source parameters were as follows: sheath gas flow rate: 50; auxiliary gas flow rate: 14; sweep gas flow rate: 3; spray voltage: 4 kV; capillary temperature: 270 °C; S-lens RF level: 45; probe heater temperature: 380 °C. The data acquisition consisted of one full MS1 scans (scan range at *m*/*z* value of 70–550, resolution at *R*_*m*/*z* 200_ = 240,000, AGC target value at 3 × 10^6^ ions, maximum IT at 500 ms) followed by MS2 scans of the most 5 abundant ions (scan range at *m*/*z* value of 200–2000, resolution at *R*_*m*/*z* 200_ = 30,000, AGC target value at 1 × 10^5^ ions, maximum IT at 54 ms). The chromatographic peak areas at MS1 level were manually integrated in Skyline. Only identifications with a mass accuracy better than 5 ppm that co-eluted with the internal standard were considered.

For IC-MS analyses of dTTP, a Dionex ICS 5000 system coupled to a Q Exactive HF mass spectrometer (both from Thermo Scientific) was used. The separation was performed on a Dionex IonPac AS11-HC column (2 mm × 250 mm, 4 µm particle size) equipped with a Dionex IonPac AG11-HC guard column (2 mm × 250 mm, 4 µm particle size) (both from Thermo Scientific) at a flow rate of 380 µl/min. The temperature of the column compartment was 30 °C. The mobile phase was deionized water and the gradient was produced by increasing the concentration of KOH. The gradient started at 10 mM KOH for 3 min, increased to 50 mM in 9 min, from 12 to 17 min KOH increased to 100 mM and was held at 100 mM from 17 to 21 min. At 21.1 min KOH concentration was decreased to 10 mM and maintained 4 min for re-equilibration. The samples were stored in the autosampler at 8 °C and 4 µl of each sample was injected onto the column. In order to connect the system to the MS, the high KOH content was removed by exchanging potassium ions against protons with a Dionex AERS 600, 2 mm anion electrolytic suppressor. The suppressor current was set to 95 mA and the device was operated at 15 °C. Water was provided to the suppressor by an external pump. The post-column makeup flow consisting of 100% MeOH, 0.1% FA had a flow rate of 120 µl/min.

The Q Exactive HF was equipped with a heated electrospray ionization source (HESI-II, Thermo Scientific) and was operated in negative ion mode. The source parameters were as follows: sheath gas flow rate: 50; auxiliary gas flow rate: 14; sweep gas flow rate: 3; spray voltage: 2.75 kV; capillary temperature: 230 °C; S-lens RF level: 45; heater temperature: 380 °C. The data acquisition consisted of one full MS scans (scan range of *m*/*z* value 400–600, at a resolution of *R*_*m*/*z* 200_ = 240,000, AGC target value 3 × 10^6^ ions, maximum IT at 500 ms) followed by 4 MS2 scans of *m*/*z* values: 480.982, 482.9916, 483.9979, 493.0096 (resolution at *R*_*m*/*z* 200_ = 30,000, AGC target value at 3 × 10^6^ ions, maximum IT at 50 ms, normalized collision energy at 38 eV).

The chromatographic peak areas at MS1 level were manually integrated in Skyline. Only identifications with a mass accuracy better than 5 ppm that co-eluted with the internal standard were considered.

### Viability assays

For viability assays, HEK293 cells (1 × 10e4 cells per well) were grown in poly-d-lysine-coated 96-well plates. LN-308 and A549 cells were grown in uncoated plates at similar densities. After 24 h of incubation, treatment with the respective chemotherapeutic drugs at the indicated concentrations occurred. To elicit the UPR, TM was applied in parallel (conc. 2.5 µg/ml), DMSO only served as a control. For the activation of HRI, BTdCPU was used, NCPdPU served as a control (both at a concentration of 10 µM). 50 µM Salubrinal was used to inhibit PP1-mediated dephosphorylation of EIF2S1. After 24 h of treatment, cell viability was determined using the CellTiter 96® AQueous One Solution Cell Proliferation Assay (Promega) according to the manufacturer’s instructions. Survival rates in presence of the indicated concentrations of the chemotherapeutic reagents were expressed in % relative to control samples that were treated with DMSO or TM only.

## Supplementary information

Supplementary Information

Peer Review File

Supplementary Data 1

Supplementary Data 2

Supplementary Data 3

Supplementary Data 4

Supplementary Data 5

## Data Availability

Raw sequencing data are accessible via Gene Expression Omnibus: GEO Series GSE129757. The mass spectrometry proteomics data have been deposited to the ProteomeXchange Consortium via the PRIDE^78^ partner repository with the dataset identifier PXD013541 and 10.6019/PXD013541. The source data underlying Figs. 1b–d, 2 and Supplementary Figs. 2, 4 and are provided in the Supplementary Data Files 2 and 3, the data underlying Fig. 3 and Supplementary Fig. 5 are provided in the Supplementary Data File 4. The data underlying Fig. 5 and Supplementary Fig. 7 are provided as a Source Data File. Source data are provided with this paper.

## Source data

source data
