# Supplementary material for: A multi-omics analysis reveals the unfolded protein response regulon and stress-induced resistance to folate-based antimetabolites
Source: Nat Commun. 2020 Jun 10;11:2936. doi: 10.1038/s41467-020-16747-y (PMC7287054; doi:10.1038/s41467-020-16747-y)

Supplementary Information

Reich et al.:

A multi-omics analysis reveals the unfolded protein response regulon and stress-induced resistance to folate-based antimetabolites

Supplementary methods

Metabolic labelling with ^{35}S-methionine

LN308 cells were grown to approx. 90% confluency before replacing the tissue culture medium by DMEM lacking Methionine and Cysteine (Sigma D0422) supplemented with 10% FCS, 2mM Glutamine (ThermoFisher), 1.5µg/ml Methionine, and 1.2µg/ml Cysteine. The medium was further supplemented with either 2.5µg/ml Tunicamycin, 1mM Cycloheximide, or DMSO (as a vehicle control). After 5min of incubation, 62.5µCi/ml [35S]Met-label (Hartmann Analytik) were added to the culture medium and incubation was continued for 2h before harvesting of the cells. Cellular extracts were prepared as described above and 50µg of total protein were subjected to SDS-PAGE using gels containing 2,2,2-Trichloroethanol (0.5%v/v, Mini PROTEAN 4-15% TGX Stain-free, BioRad). After UV-irradiation, proteins were visualized using a ChemiDoc Imaging System (BioRad). Subsequently the gel was incubated in Amplify solution (GE Healthcare) and dried. ^{35}S-Methionine and -Cysteine incorporation was visualized and quantified using a Personal Molecular Imager (BioRad).

Growth in serine-depleted medium

LN-308 cells were cultured in DMEM (supplemented with 10% FCS) for 18h. After aspiration of the medium, cells were washed with PBS and grown for 24h in MEM (supplemented with 10% dialyzed FBS, and 200µM L-glutamine) lacking serine. In control samples cultured in parallel, L-serine was supplemented at a concentration of 400µM. Treatment with 2.5µM TM or DMSO as control occurred in parallel. Cell viability was assayed using the CellTiter 96® AQueous One Solution Cell Proliferation Assay (Promega) according to the manufacturer's instructions.

Sampling overview:

Experiment	Incubation [h]	Control	ER stress induced
Proteomics	0	DMSO (n=3)	DMSO (n=3)
	6	DMSO (n=3)	Tunicamycin (n=3)
	16	DMSO (n=3)	Tunicamycin (n=3)
	24	DMSO (n=3)	Tunicamycin (n=3)
	6	DMSO (n=3)	Thapsigargin (n=3)
	16	DMSO (n=3)	Thapsigargin (n=3)
	24	DMSO (n=3)	Thapsigargin (n=3)
Expression profiling	0	control (n=3)	
	2		Tunicamycin (n=3)
	6		Tunicamycin (n=3)
	2		Thapsigargin (n=1)*
	6		Thapsigargin (n=1)*
Ribosome Profiling	0	control (n=3)	
	2		Tunicamycin (n=3)
	6		Tunicamycin (n=3)
	2		Thapsigargin (n=1)
	6		Thapsigargin (n=1)

* analysis performed with technical replicates of a biological experiment

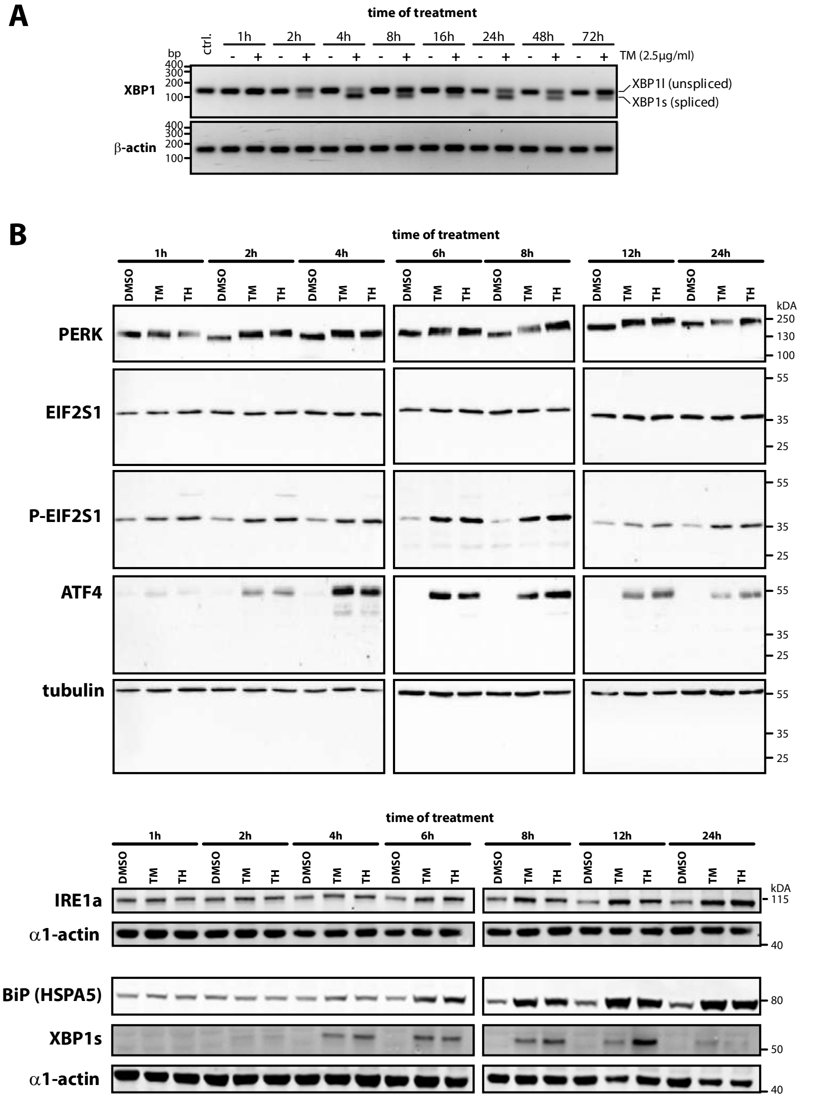

Supplementary Figure 1: Regulation of known UPR targets upon stimulation of LN308 cells with TM or TH

A: RT-PCR analysis of IRE1-mediated, cytoplasmic processing of XBP1 mRNA (upper panel) after continuous stimulation with 2.5µg/ml of TM (lanes denoted +) for the indicated durations. Control reactions were performed in parallel using DMSO only (lanes denoted -). The unprocessed XBP1l mRNA (unspliced) and the processed XBP1s mRNA (spliced) are indicated on the right. β-actin MRNA served as a control (lower panel). Molecular weight markers are given on the left. Shown are representative gels of 3 biologically independent experiments.

B: Western Blotting analyses of UPR-mediated regulation after treatment with either TM or TH for various durations (as indicated at the top). First set of blots from top to bottom: analysis of (1) PERK phosphorylation as indicated by slightly reduced gel mobility, (2) total EIF2S1 protein, (3) phosphorylation of Ser51 of EIF2S1, (4) ATF4 protein expression, and (5) tubulin protein levels as a loading control. Second set of blots from top to bottom: analysis of (1) IRE1 protein level, (2) α1-actin as the corresponding loading control, (3) BiP (HSPA5) protein expression, (4) XBP1 protein expression, and (5) the corresponding α1-actin loading control. Molecular weight markers in kDa are indicated on the right. Shown are representative blots of 3 or more biologically independent experiments.

Uncropped gels and Western Blot membranes are provided in the Source Data file.

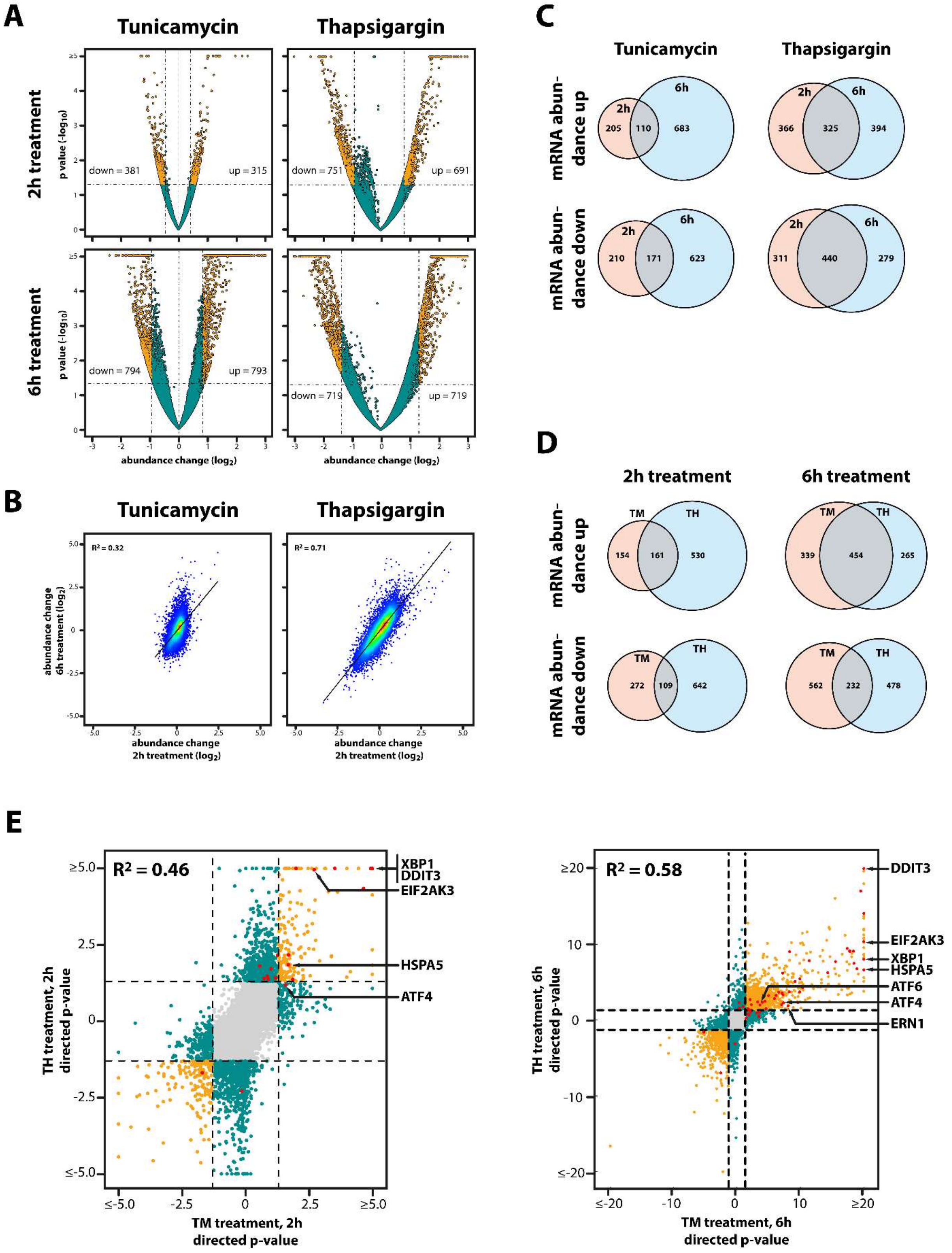

Supplementary Figure 2: Transcriptomic changes upon induction of the UPR

A: Volcano plots of transcriptomic changes after treatment of LN308 cells with either TM or TH for 2h or 6h. Numbers of statistically significant up- or down-regulated genes are

indicated in each plot. **B-E:** Comparison of RNA abundance changes induced upon exposure of LN308 to TH or TM for 2h or 6h. Scatter plots and venn diagrams that compare changes at different time points are shown in panels B and C. Comparisons of the treatment-specific changes to RNA abundance are shown in panels D and E. In panel B correlation coefficients for the comparisons are given in the top left corner. In each venn diagram the numbers of statistically significant up- and down-regulated mRNA species are given. Color-coding of the scatter plot in panel E as described for Figure 1, mRNAs encoding select factors involved in the UPR are highlighted by arrows. Directed *p*-values are defined as -log10 (*p*) times the direction of the effect. Plotted are averaged values.

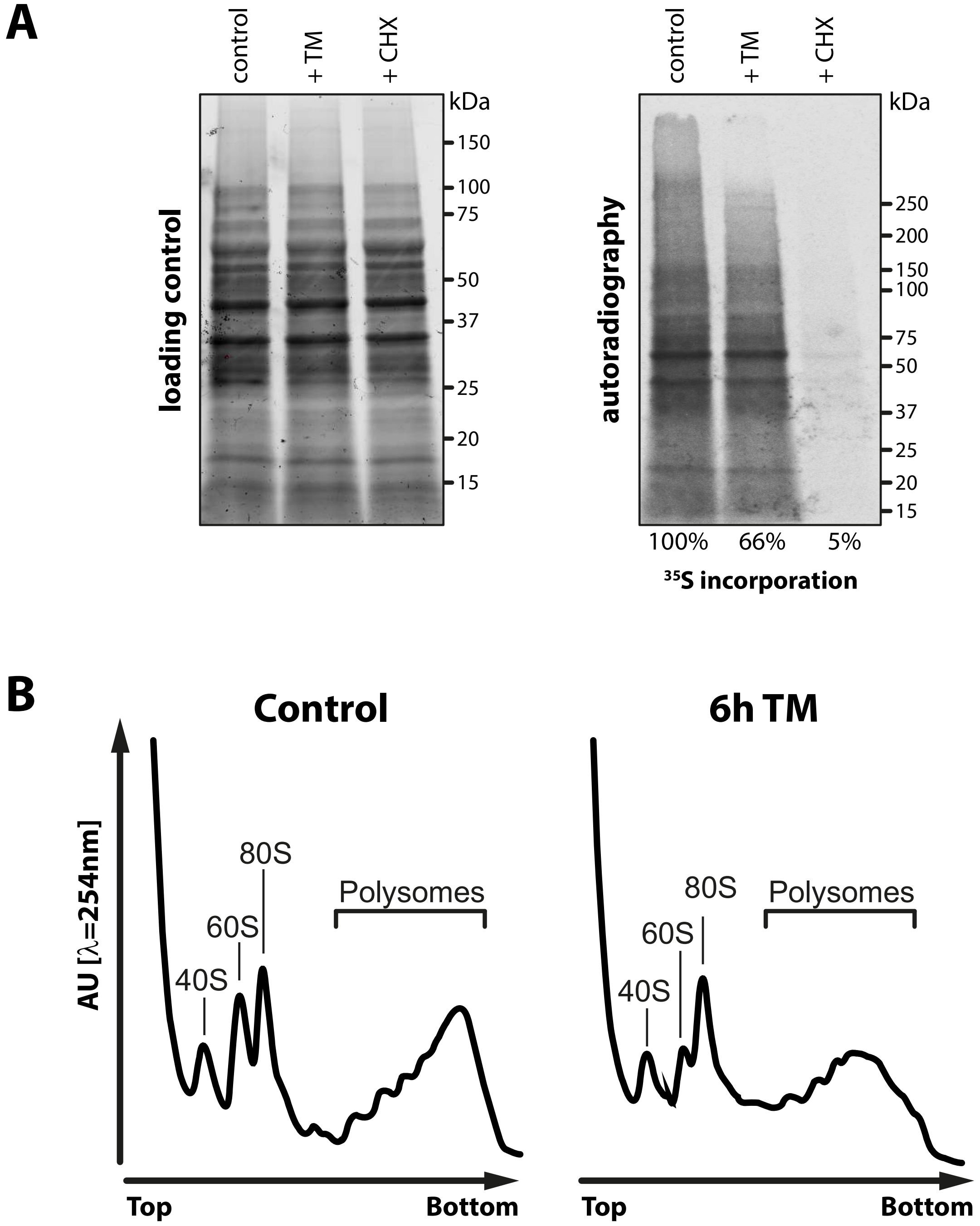

Supplementary Figure 3: Reduction of translation upon treatment with Tunicamycin (see also Figs 1D and 2B)

A: Incorporation of ^{35}S-labelled methionine in TM-treated LN308-cells. Loading control on the left (stained with 2,2,2-Trichloroethanol), autoradiography of the same gel on the right. Inhibition of translation with Cycloheximide served as control. Incorporation rates of ^{35}S-Met (in % relative to the DMSO-treated control) are given below the lanes. Molecular weight markers are indicated on the right. **B:** Analyses of translation complexes by polysome profiling of control-treated (left panel) and TM-treated LN308 cells (right panel). Positions of free 40S and 60S subunits as well as 80S monosomes and polysomes are indicated. Representative data of at least three biologically independent experiments are depicted.

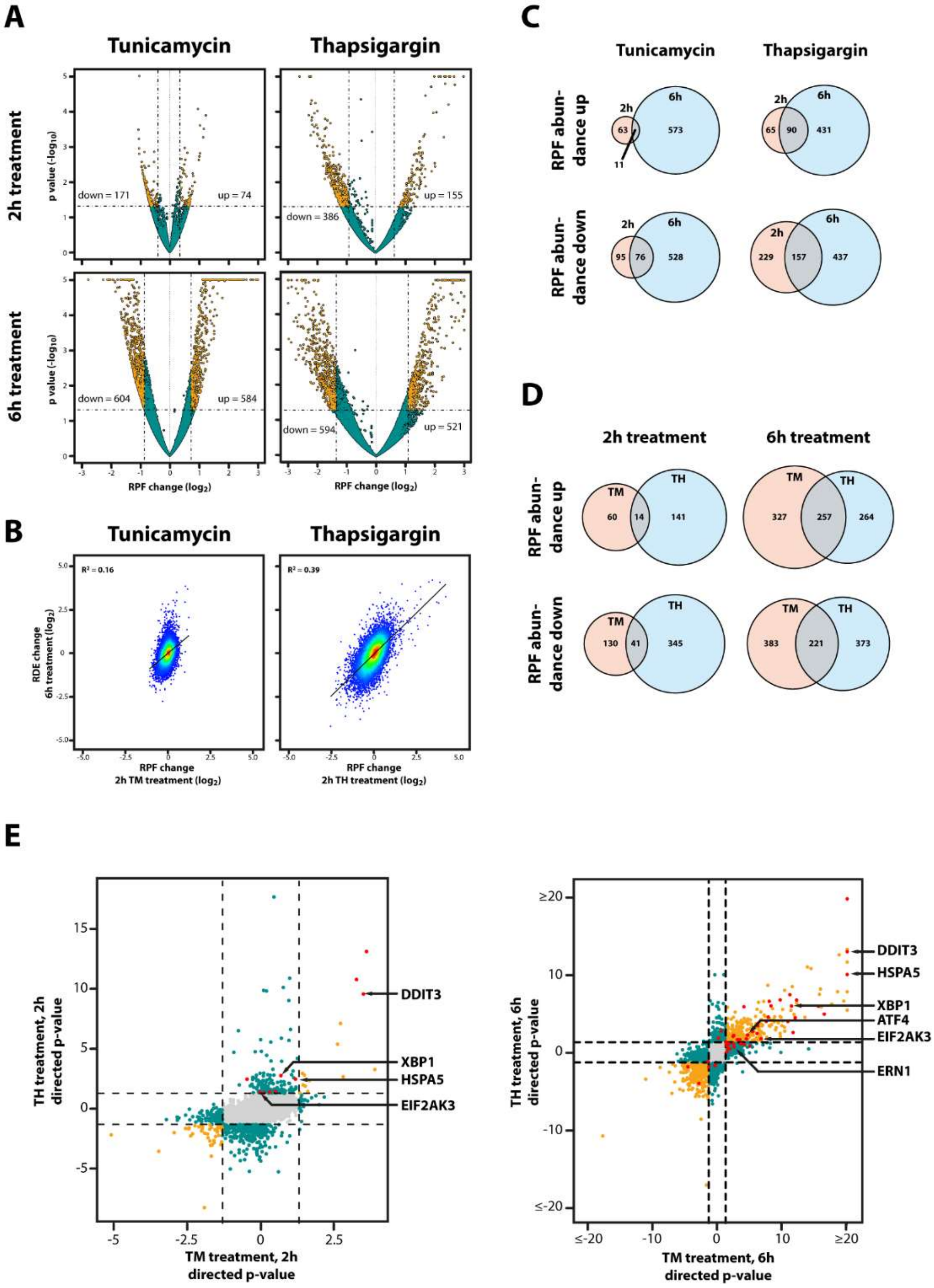

Supplementary Figure 4: Changes in RPF abundance upon induction of the UPR (see also Figs 1D and 2B)

Similar to figure S2, except that changes in the abundances of ribosome-protected fragments (as determined by ribosome profiling) are plotted 2h and 6h after treatment with either TM or TH. Plotted are averaged values.

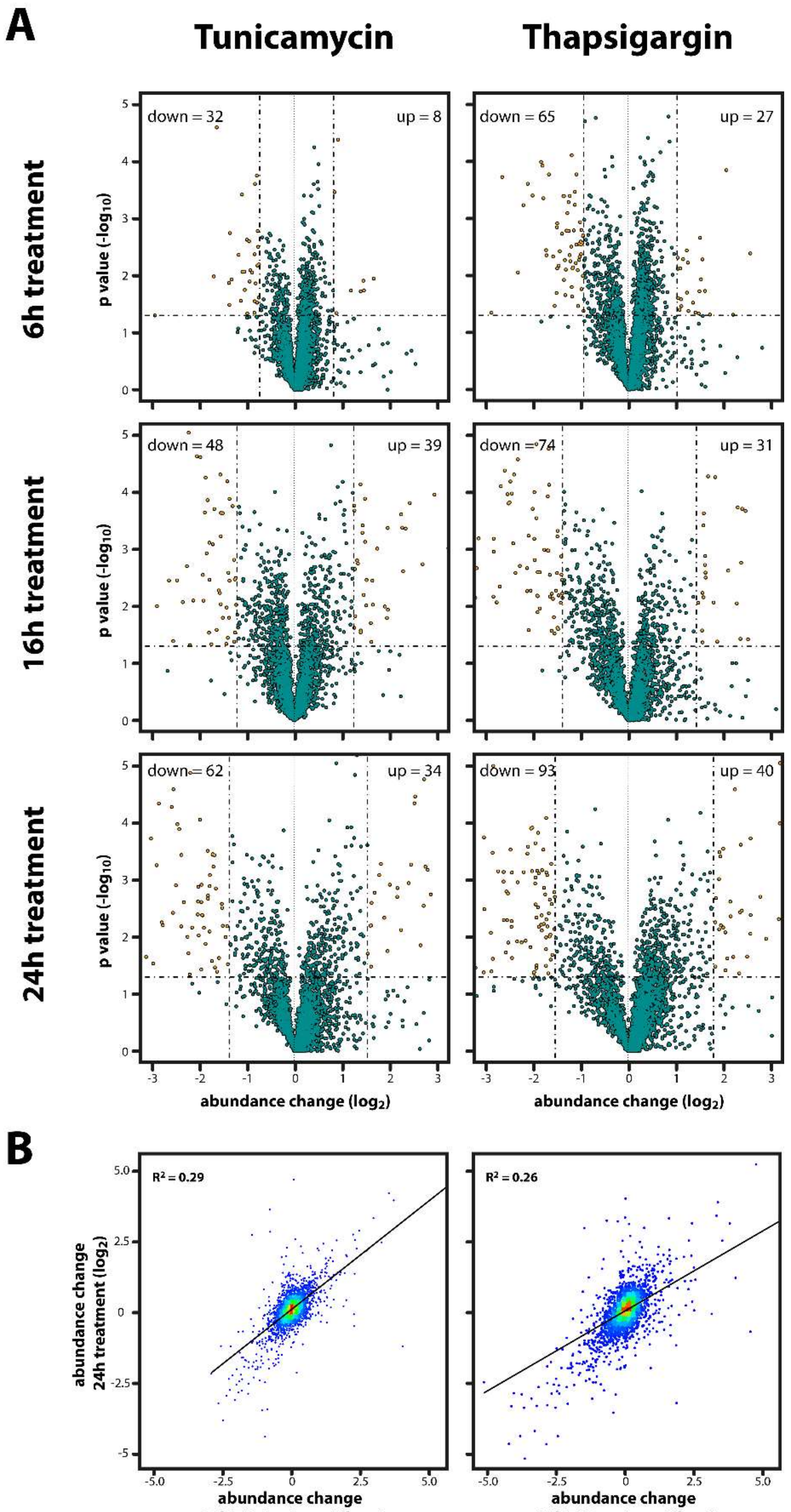

Supplementary Figure 5: Changes in protein abundance upon induction of the UPR (see also Fig 3)

A: Volcano plots depicting changes in protein abundance after treatment with TM or TH for 6h (top), 16h (middle), or 24h (bottom). **B:** Comparison of changes to the proteome 16h and 24h after stimulation with either TM or TH. Correlation coefficients are given in the top left corner of each diagram. Plotted are averaged values.

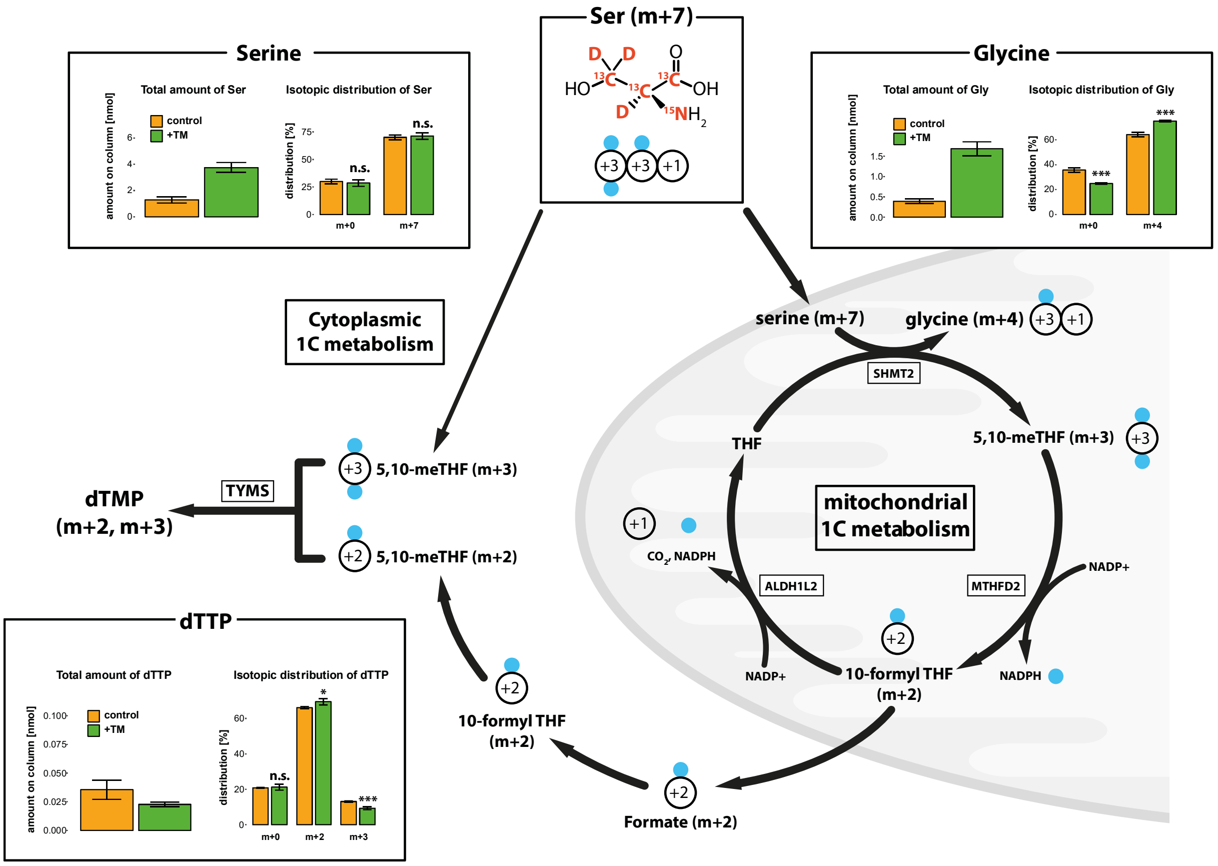

Supplementary Figure 6: Metabolic analysis with a labelled serine tracer (see also Fig 5)

Overview of metabolic labelling using stable isotope-labelled serine (depicted at the top, labelled atoms highlighted in red; schematic representation shown below with each deuterium depicted as blue dot and each carbon unit as circle with the additional masses per carbon unit displayed). Biochemical turnover of serine via the mitochondrial folate-based 1C metabolism is depicted schematically (see also Fig 5). Cytosolic synthesis of dTMP by the enzyme TYMS is depicted on the left; note that its substrate carbon units generated via the cytoplasmic 1C metabolism and the mitochondrial pathway differ in one mass unit. Inserts: quantification of total serine, glycine and dTTP levels and the isotopic mass distributions after 24h of labelling in non-stressed cells (yellow bars) or in cells subjected to TM treatment (green bars).

Data are represented as mean ± SD of 3 biologically independent experiments. Student's t-test was used for the statistical testing with the following parameters: two-sided, true variance equality and confidence level at 0.95. No adjustments for multiple comparisons were made. *P*-values: Serine M+0 0.590, Ser M+7 0.590, Glycine M+0 0.001, Glycine M+4 0.001, dTTP M+0 0.713, dTTP M+2 0.038, and dTTP M+3 0.002. Abbreviations: n.s. not significant p>0.05, * p<0.05, *** p<0.005. Source data are provided as a Source Data file.

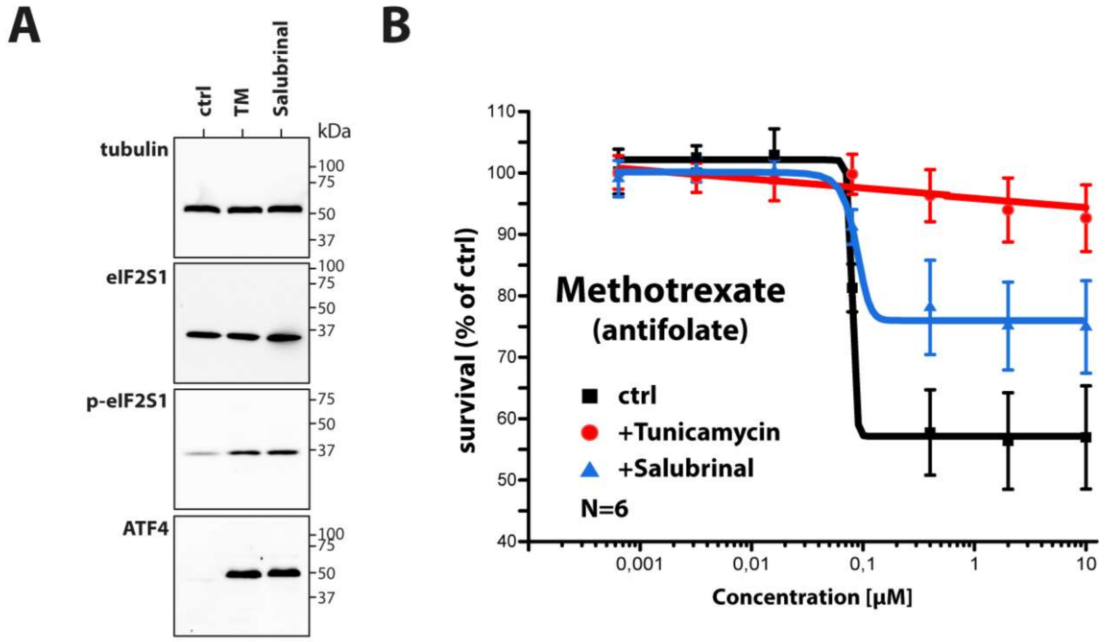

Supplementary Figure 7: Inhibition of EIF2S1 dephosphorylation induces partial resistance to MTX in cultured LN308 cells (see also Fig 6)

A: Western blotting against tubulin, EIF2S1, Ser51 phosphorylated EIF2S1, and ATF4 of LN308 cells treated for 24h with Salubrinal, TM, or DMSO as control (as indicated at the top). Molecular weight markers are shown on the right. Representative blots of three biologically independent experiments are depicted. Uncropped Western Blot membranes are provided in the Source Data file. **B:** Survival of LN308 cells after 24h of treatment with the indicated concentrations of MTX in the absence presence of Tunicamycin (red), Salubrinal (blue), or of control cells (black, vehicle control). Data are represented as mean ± SD of 6 biologically independent experiments.

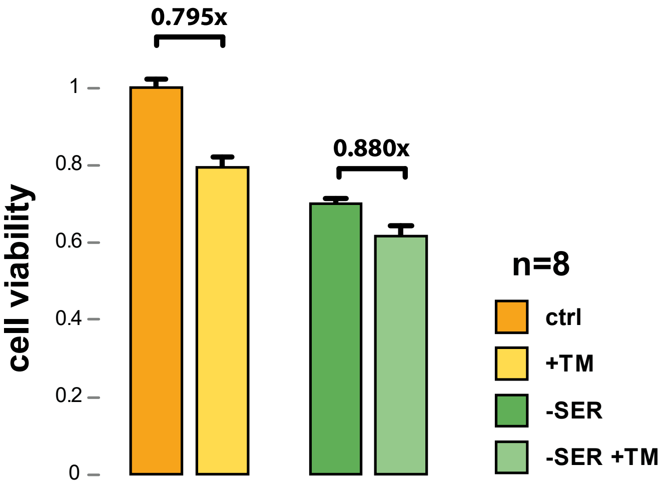

Supplementary Figure 8: Cell viability in serine-depleted medium
Cell viability in serine-depleted medium (green bars) and serine-supplemented medium (yellow/orange bars). Growth was analyzed by an MTS assay 24h after continuous treatment with 2.5µM TM (light bars) or DMSO as a control (dark bars). Depicted are mean values ±SD of 8 technical replicates.

Supplementary information: uncropped images of RT-PCR gels and Western Blot membranes

Figure 4A

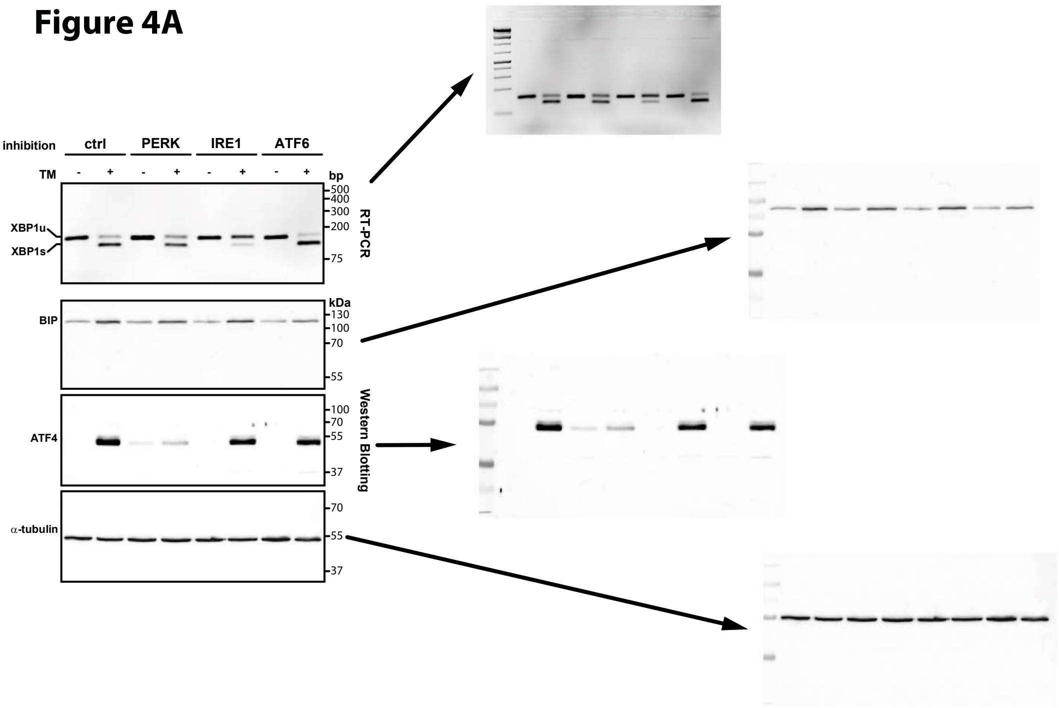

Figure 4B

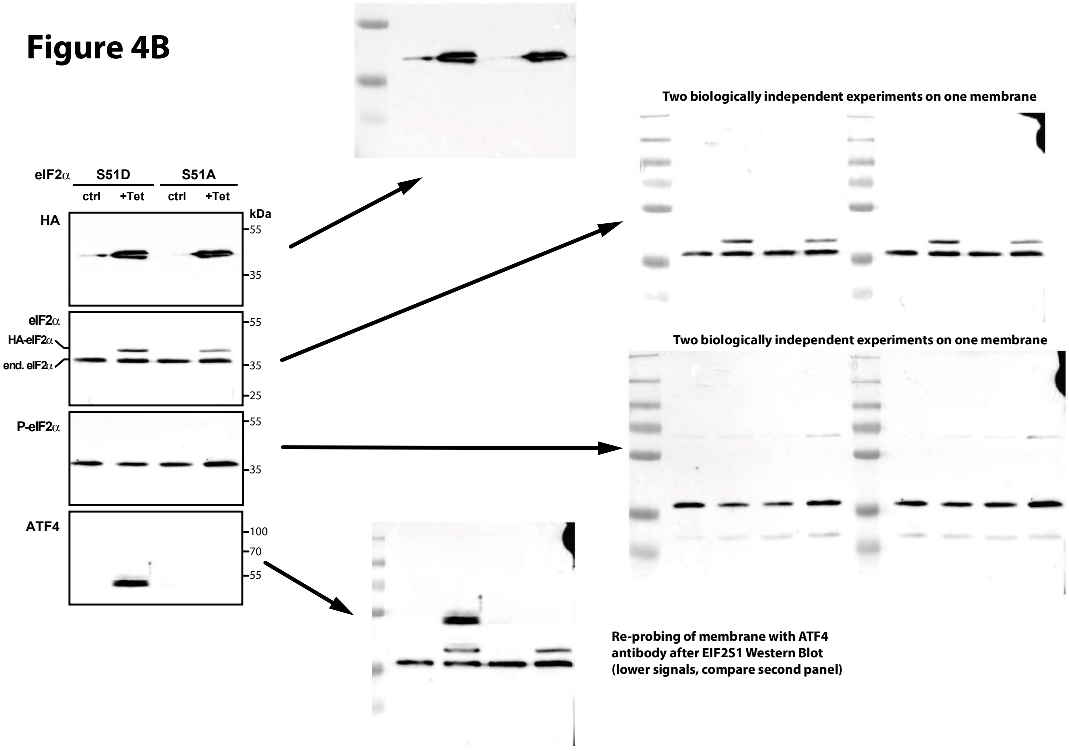

Figure 4C

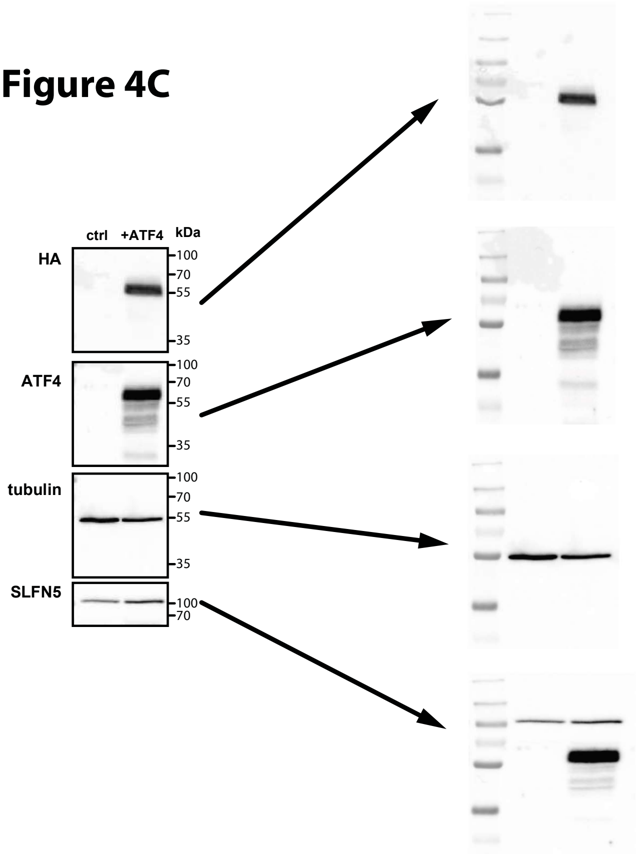

Figure 6D

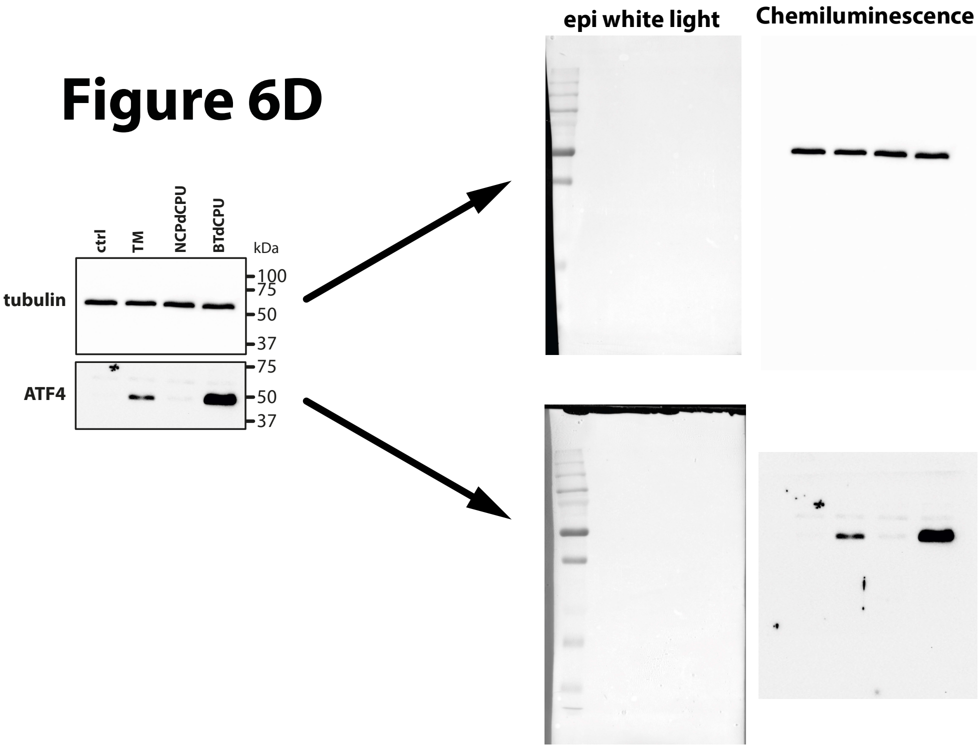

Suppl. Figure 1A

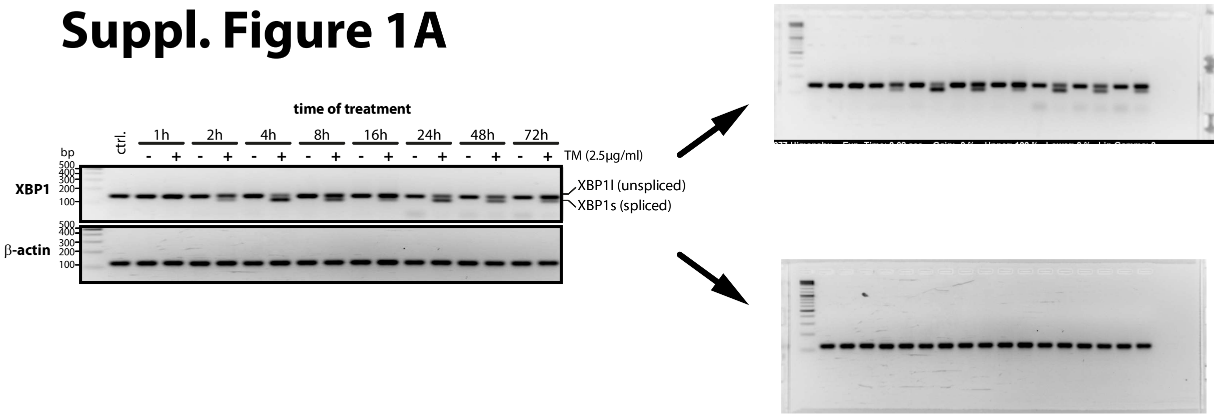

Suppl. Figure 1B

upper panels, ECL detection

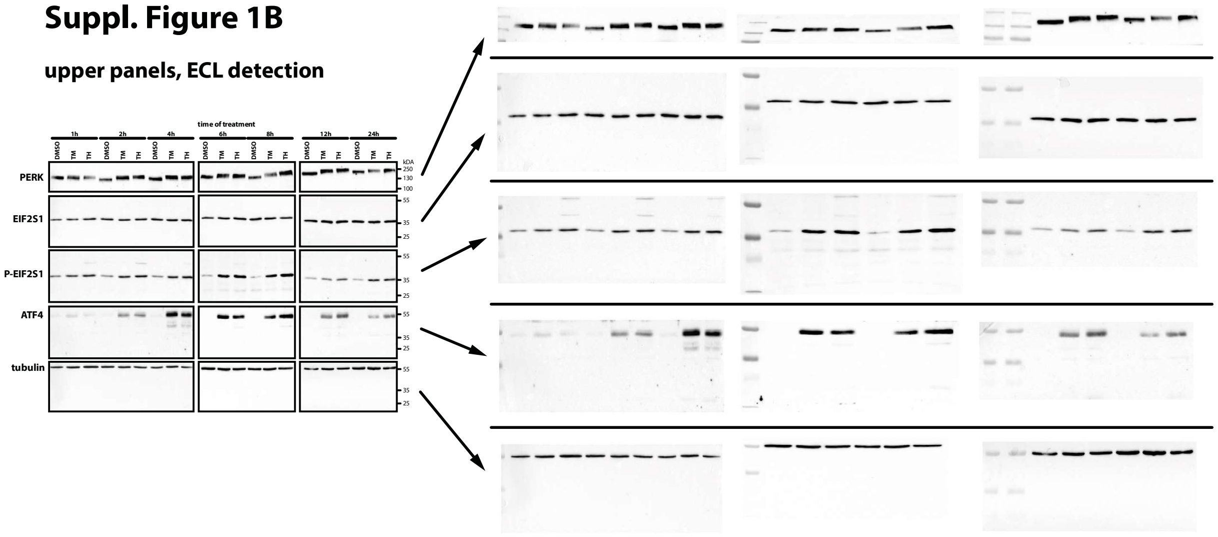

Suppl. Figure 1B

lower panels, IRDye detection

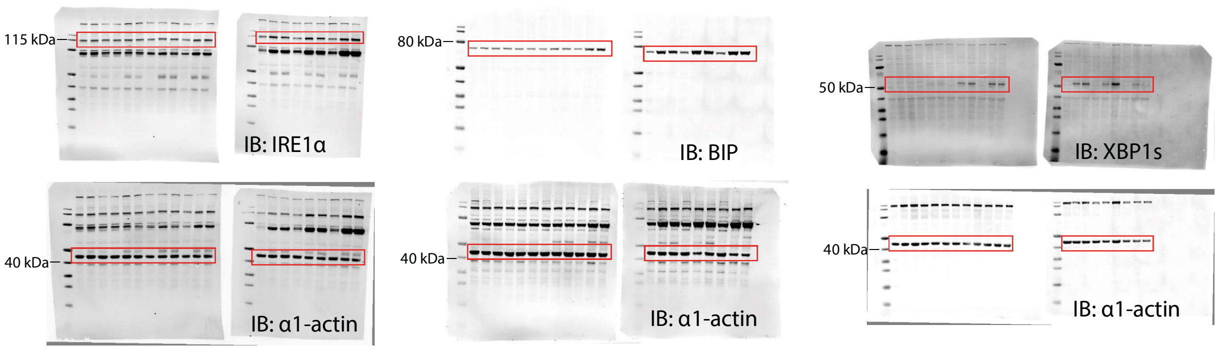

Suppl. Figure 7A

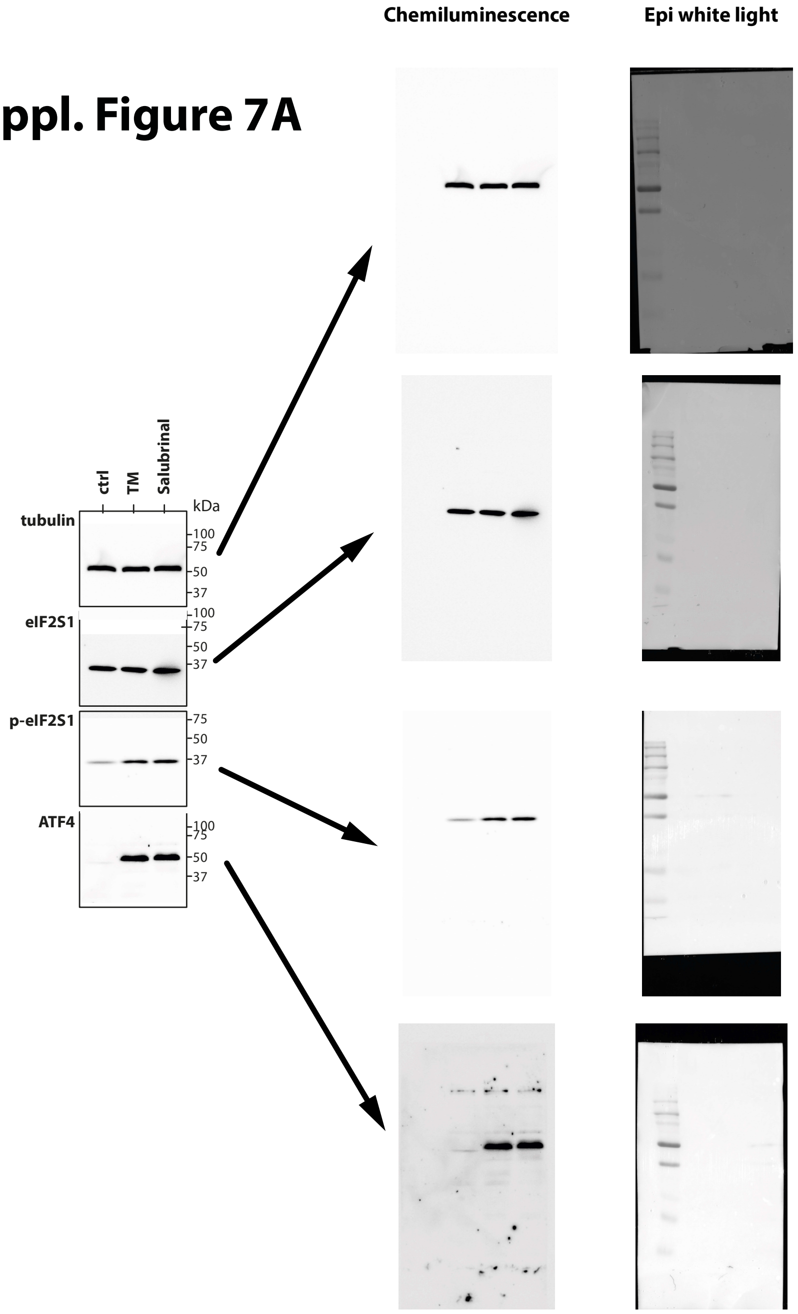

Supplement: Supplementary file 1 — Supplementary Information [file 41467_2020_16747_MOESM1_ESM.pdf]
