# Supplementary material for: A multi-omics analysis reveals the unfolded protein response regulon and stress-induced resistance to folate-based antimetabolites
Source: Nat Commun. 2020 Jun 10;11:2936. doi: 10.1038/s41467-020-16747-y (PMC7287054; doi:10.1038/s41467-020-16747-y)
Supplement: Supplementary file 2 — Peer Review File [file 41467_2020_16747_MOESM2_ESM.docx]

Peer Review Information

**Journal:** Nature Communications

**Manuscript title:** Anti-folate resistance and metabolic reprogramming induced by the unfolded protein response

**Corresponding author name(s):** Dr Jan Mednbach

**Editorial notes:**

**Transferred manuscripts**

This manuscript has been previously reviewed at another journal that is not operating a transparent peer review scheme. This document only contains reviewer comments and rebuttal letters for versions considered at *Nature Communications*.

**Transferred manuscripts (no peer review at *Nature Communications*)**

This manuscript has been previously reviewed at another journal that is not operating a transparent peer review scheme. The manuscript was considered suitable for publication without further review at *Nature Communications*.

**Redactions – transferred manuscripts (mention of previous referee reports from elsewhere)**

This manuscript has been previously reviewed at another journal that is not operating a transparent peer review scheme. This document only contains reviewer comments and rebuttal letters for versions considered at *Nature Communications*. Mentions of prior referee reports have been redacted.

**Redactions – transferred manuscripts (mention of the other journal)**

This manuscript has been previously reviewed at another journal that is not operating a transparent peer review scheme. This document only contains reviewer comments and rebuttal letters for versions considered at *Nature Communications*. Mentions of the other journal have been redacted.

**Redactions – unpublished data**

Parts of this Peer Review File have been redacted as indicated to maintain the confidentiality of unpublished data.

**Redactions – confidential patient information**

Parts of this Peer Review File have been redacted as indicated to maintain patient confidentiality.

**Redactions – published data**

Parts of this Peer Review File have been redacted as indicated to remove third-party material where no permission to publish could be obtained.

**Amendments – published data**

[Figure X/The figure on page X] in this Peer Review File has been amended to remove third-party material where no permission to publish could be obtained.

**Published data (credit line for image)**

[Figure X/The figure on page X] in this Peer Review File is reproduced with permission from [Copyright Holder].

**Redactions – reviewer opt-out**

Parts of this Peer Review File have been redacted as indicated as we could not obtain permission to publish the reports of reviewer ###.

**Reviewer comments in marked-up manuscript**

In their review of the [first/second/third/...] version of this manuscript, reviewer ### added their comments to the manuscript file. These comments, excluding minor textual revisions, have been copied into this Peer Review File.

**Reviewer comments & decisions:**

**Reviewer comments, first version:**

Reviewer #1 (Remarks to the Author):

The manuscript by Reich et al. presents an in-depth analysis of time-resolved gene expression changes as the astrocytoma cell line LN-308 responds to unfolded protein stress triggered by either Tunicamycin or Thapsigargin. Regular RNA-seq is used to probe changes in (m)RNA abundance, ribosome profiling to measure changes in mRNA translation state and mass spectrometry to assess changes in protein abundance. Documenting the various changes at the different levels of gene expression confirms expectations based on much more limited prior studies and provides more comprehensive data as a useful novel resource. For example, a 267-member strong UPR regulon is defined that extends well beyond genes previously implicated in the pathway. Picking up on an increased expression of enzymes involved in channelling intermediary metabolites from glycolysis to folate-mediated one-carbon metabolism, the authors further describe a UPR-induced resistance to folate-based antimetabolite drugs, which is of particular interest to the use of these drugs in cancer treatment. The study, in its extent and the specific follow-up that was pursued, is novel and of clear interest for research into cellular stress responses as well as cancer biology. New impulses to research in these areas are expected to arise from it. The work is carefully done, very clearly presented and, therefore, convincing (but see my suggestions for improvement below).

In general, I commend the authors for their efforts to prepare clear figures and their use of statistical analyses throughout. Nevertheless, there are certain areas of the manuscript where I have not been able to fully grasp how biological replicates were used to generate graphs and exactly what filtering parameters were used to enumerate or colour-code significantly altered genes etc.

Most importantly, the authors should include a simple table to indicate the number of biological replicates that were performed for each of the omics-type analyses. Something like a tabular inventory of datasets for those that do not require downloading the raw data. I quickly checked the GEO deposition and found that most sequencing-based data was done in triplicate but it seems that this might be true only for one of the inhibitors, and not the other. If true, this would be of some concern. Additionally, the figure legends to figures showing the various scatter and volcano plots could state if data from replicates were merged (averaged, summed up) for the analyses. I am also a little confused about EXACTLY what criteria were used to filter for significance. The methods section states that an FDR of 5% was applied as well as a 95% confidence interval (isn’t that redundant?). But the appearance of some figures (e.g. Figure 1C,D) suggest to me that a fold-change criterion was also used. There is also evidence that a ‘dynamic’ filter for fold-change is used for some of the sequencing data (e.g. Figure S2A, S3A). This might be based on a strategy similar to the one used for the proteomics data (median log2 fold-change ±twice the SD; e.g. Figure S4A). The methods section should be expanded to cover this and it might also be best to explicitly state the key aspects of this in each figure legend. The various Western blot panels shown throughout look convincing but the legends should indicate if these results were representative of replicate experiments.


Reviewer #2 (Remarks to the Author):

The manuscript by Reich et al. seeks to understand the role of the endoplasmic reticulum unfolded protein response (UPR) in anti-folate resistance and metabolic reprogramming in astrocytoma. They suggest that chemical induction of the UPR controls a set of genes encoding enzymes that diverts metabolites from glycolysis to fuel mitochondrial folate‐based one‐carbon metabolism. Moreover, they suggest that the UPR contributes to chemoresistance of FDA approved folate-based anti-metabolites. Although the findings of the manuscript are original and interesting, there are major shortcoming in this manuscript. In particular further experimental evidence is required to support the title and claims in the manuscript. Please find suggested major and minor comments below:
Major comments
1. More than half of the main figures in the manuscript are focused on transcriptional, translational and proteomic analysis in LN308 astrocytoma cells after chemical induction of the UPR. Therefore, the title does not reflect the majority of the data in the manuscript. I suggest considering a revision of the title, as this manuscript mainly focuses on the kinetics of the transcriptome, translatome and proteome after chemical induction of the UPR, with minor focus on mitochondrial 1c metabolism and folate resistance.
2. The studies are carried out in one cell line and confirmatory experiments should also be carried out in other cell lines.
3. The choice of cell model is not clear. In the manuscript the authors have referred to breast cancer several times. Many tumor cells/ cell lines, including breast cancer cells lines have basal UPR activity. It would be interesting to see the effect basal UPR on mitochondrial 1C metabolism and reversal by inhibiting PERK activity.
4. The authors have not sufficiently shown that LN308 cells switch to mitochondrial 1C metabolism after UPR induction. Assessing the reliance of these cells for serine metabolism vs glycolysis should be examined. For example, monitoring proliferation of cells in serine depleted media after UPR activation/deletion of UPR components. Moreover, serine uptake and NADPH production in the mitochondria should be assessed after knockdown of PERK/p-eIF2α/ATF4 to link PERK directly to mitochondrial 1c pathway. Serine labelling can also demonstrate electron transfer through the mitochondrial 1c pathway.
5. To support the in vitro experiments performed in figure 5, in vivo studies of astrocytoma cell models should be completed to examine tumour volume and UPR activation post anti-folate treatment to examine chemoresistance. PERK should be linked to anti-folate resistance by using PERK inhibitor or PERK knockout cells after folate treatment.
6. The experiment in figure 4A using pharmacological inhibitors should be further validated using siRNA/shRNA techniques against IRE1, PERK and ATF6.
7. The effect the authors attribute to UPR seems to be mediated by the PERK/eIF2 α/ATF4 pathway and this is a common pathway with ISR. The authors do not see a role for ATF6/IRE1 in their studies. As such, in line to what the authors have eluded to on page 15 and 16, ISR rather than UPR may be responsible for metabolic reprogramming. I believe that the authors need to make this distinction and to determine the role of other eIF2a kinases in the regulation of the pathway. Such data would significantly increase the impact of the study.
8. Do anti-folate compounds induce UPR/ISR?

Minor comments
1. The qPCRs in Figure 4 are not well labelled, particularly the bottom left graph. It is also not clear from reading the figure legend what treatments were performed in this particular experiment. I also suggest changing the y-axis label to mRNA abundance/expression rather than mRNA induction.
2. Some figure legends describe the results rather than describing the experimental information, for example figure 4 ‘Expression of a phosphomimetic eIF2a protein (lanes S51D), but not a non‐phophorylatable variant (lanes S51A), increases SLFN5, SHMT2, and MTHFD2 mRNA abundance in HEK293 cells’
3. Does the treatment with TM and TH induce cell death? What was % cell death at each sampling time point?


Reviewer #3 (Remarks to the Author):

This manuscript reports on cellular stress response pathways that modify responses to chemotherapeutic drugs. Deep sequencing analysis of transcription and translation are combined with proteomics in order to characterize short- and long-term responses to mechanistically distinct inducers of the ER UPR. Joint analysis of these data reveal a core UPR regulon that includes known targets of this well-studied pathway as well as the complete pathway for folate-based 1-carbon metabolism. Notably, folate is a target for anti-cancer chemotherapy, and UPR induction provides dramatic resistance to folate antimetabolite drugs.

This study uncovers additional targets of the UPR / integrated stress response pathway and demonstrates a clear impact on cell metabolism and physiology with likely clinical relevance. As such, it is likely to attract broad interest. Overall the data are clear and support the key conclusions, though I have specific concerns with some interpretations as described below.

1. Ribosome profiling and proteomic mass spectrometry data are used to argue for a general reduction in translation. For example, at the top of p. 7 it is written that “we find…an overall reduction from RPFs from numerous loci…” and on p. 8 that proteomics results are “…indicative of a globally reduced protein synthesis rate.” However, both approaches suffer from a global normalization problem that is not addressed in this manuscript. A ribosome profiling experiment reports on the overall pool of translating ribosomes and is insensitive to changes in the bulk number of translating ribosomes (e.g., Liu et al., Cell Systems 2017). Likewise, the mass spectrometry data captures the fractional abundance of individual proteins in the overall proteome. Neither approach can directly assess changes in bulk translation without careful use of spike-in standards.

Alternate techniques, such as pulse labeling with OP-Puro or L-AHA permit analysis of bulk protein synthesis.

2. The manuscript cites Andreev et al., eLife 2015 in discussing ISR translation, but not Sidrauski et al., eLife 2015, which seems at least as relevant.

3. The manuscript should compare ISR translation targets with those reported in Andreev et al. or in Sidrauski et al. Sidrauski et al. use tunicamycin, one of the UPR inducers here, whereas Andreev et al. use arsenite; each comparison would add to the analysis.

4. The MTX resistance data are quite striking, but tests linking this to the ISR are performed in a different cell line (HEK293). It seems important to show that MTX resistance depends on UPR / ISR signaling specifically in transformed cells. Furthermore, the drugs ISRIB and salubrinal provide a straightforward way to manipulate the ISR that can be applied in many different cell types without transfection or transduction. Furthermore, results using these drugs can translate clinically in a way that transgenic expression of phosphomimetic proteins cannot.

5. The IC50 is 5FU is said to remain unaffected (p. 14 and Figs 5B and C) after UPR induction, but it seems there is actually a modest but real increase in resistance.

6. as a minor point, at the top of p. 12, “or” in the list of ATF4-responsive genes should be “and”.

**Author rebuttal, first version:**

**Reviewer #1 (Remarks to the Author):**

The manuscript by Reich et al. presents an in-depth analysis of time-resolved gene expression changes as the astrocytoma cell line LN-308 responds to unfolded protein stress triggered by either Tunicamycin or Thapsigargin. Regular RNA-seq is used to probe changes in (m)RNA abundance, ribosome profiling to measure changes in mRNA translation state and mass spectrometry to assess changes in protein abundance. Documenting the various changes at the different levels of gene expression confirms expectations based on much more limited prior studies and provides more comprehensive data as a useful novel resource. For example, a 267-member strong UPR regulon is defined that extends well beyond genes previously implicated in the pathway. Picking up on an increased expression of enzymes involved in channelling intermediary metabolites from glycolysis to folate-mediated one-carbon metabolism, the authors further describe a UPR-induced resistance to folate-based antimetabolite drugs, which is of particular interest to the use of these drugs in cancer treatment. The study, in its extent and the specific follow-up that was pursued, is novel and of clear interest for research into cellular stress responses as well as cancer biology. New impulses to research in these areas are expected to arise from it. The work is carefully done, very clearly presented and, therefore, convincing (but see my suggestions for improvement below).

In general, I commend the authors for their efforts to prepare clear figures and their use of statistical analyses throughout.

*Thank you very much!*

Nevertheless, there are certain areas of the manuscript where I have not been able to fully grasp how biological replicates were used to generate graphs and exactly what filtering parameters were used to enumerate or colour-code significantly altered genes etc.

Most importantly, the authors should include a simple table to indicate the number of biological replicates that were performed for each of the omics-type analyses. Something like a tabular inventory of datasets for those that do not require downloading the raw data.

*We agree with the reviewer and now include a tabular summary of the analyzed samples in the supplementary information.*

I quickly checked the GEO deposition and found that most sequencing-based data was done in triplicate but it seems that this might be true only for one of the inhibitors, and not the other. If true, this would be of some concern.

*We thank the reviewer for pointing this out! Indeed, we analyzed fewer samples for treatment with Thapsigargin. While we generated three biological replicates to analyze the effects of Tunicamycin treatment (3 biological replicates per treatment condition with 2 technical replicates for expression profiling), we only have technical replicates for the analysis of effects mediated by Thapsigargin. In the case of expression profiling, we performed 22 paired-end RNAseq experiments in total. The reads from technical replicates have been summed up for further analyses which is why there is only a single dataset for each individual treatment condition in the GEO entry (we think it is fair to provide a single dataset instead of different files since the technical replicates do not reconcile the biological variability).*

*Based on these data, gene level expression was quantified using HTSeq (Anders et al. 2015) and raw RNA-sequencing count data normalized and fold changes computed using the DESeq2 library with the default DESeq function (Love et al. 2014). In brief, the gene ranking is based on stable estimation of effect sizes (LFC) per gene. The read counts for a single gene were assumed to follow a negative binomial distribution with mean and dispersion parameter. A generalized linear model (GLM) for each gene was fitted using the count matrix. Applying the GLM fitting, maximum likelihood estimates (MLE) of the dispersion parameter were obtained for each LFC. Additionally, fitting a zero-centered normal distribution for all MLEs was applied to get a standard error for each estimate. In a second GLM turn, this distribution was used to estimate maximum a posteriori as the final estimate. These shrunken LFCs were used with the standard errors for testing. To test the hypothesis for differential expression, a Wald test (using single parameter testing) was utilized for each gene. Afterwards, p-values were adjusted for multiple testing using the procedure of Benjamini and Hochberg.*

*We fully agree with the reviewer that under-sampling of the biological variability might be of some concern for the Thapsigargin-treated samples, however, we have used this data only to validate candidate gene lists that were generated by analyses of Tunicamycin-mediated changes to gene expression with 3 biological and 2 technical replicates per treatment condition. Due to its limited statistical power, the Thapsigargin dataset therefore was not used to expand our candidate lists, but rather for subtractive analyses with the aim to remove potential false positive loci from our candidate list of genes that are potentially regulated by the UPR. It is likely that the analyses of additional Thapsigargin-treated samples would rather extend our UPR-regulon (by validation of additional gene loci) instead of eliminating further candidates.*

*We believe that the strong enrichment and the robust identification of previously known UPR-target genes validates the procedure. Moreover, we state in our manuscript that the UPR regulon – the list of candidate genes that are very likely induced by the UPR – requires independent experimental validation, which we have performed in part. We have analyzed the expression of select candidate genes to biochemically and genetically confirm that their expression is under the control of UPR-mediated signaling.*

*We have, however, realized that we have not explicitly explained the procedure of how we statistically dealt with the limited number of Thapsigargin samples in the original version of the manuscript – a shortcoming, which we have now corrected by extending the methods section of the manuscript. We again thank the reviewer for bringing this to our attention!*

Additionally, the figure legends to figures showing the various scatter and volcano plots could state if data from replicates were merged (averaged, summed up) for the analyses.

*Replicates of these datasets were averaged. The figure legends have been updated accordingly.*

I am also a little confused about EXACTLY what criteria were used to filter for significance. The methods section states that an FDR of 5% was applied as well as a 95% confidence interval (isn’t that redundant?).

*For the proteomics analysis we applied a FDR of 1% for peptide and protein identification. 95% confidence interval was used as quantification criteria. These steps are related however not in a way to produce redundancy.*

But the appearance of some figures (e.g. Figure 1C,D) suggest to me that a fold-change criterion was also used. There is also evidence that a ‘dynamic’ filter for fold-change is used for some of the sequencing data (e.g. Figure S2A, S3A). This might be based on a strategy similar to the one used for the proteomics data (median log2 fold-change ±twice the SD; e.g. Figure S4A). The methods section should be expanded to cover this and it might also be best to explicitly state the key aspects of this in each figure legend.

*In all cases, mean values are plotted derived from the individual software packages (e.g. DESeq2). This information is now included in the individual figure legends.*

*In Figure 1C and D, two criteria were used to identify ‘regulated’ genes: (a) a fold change in the 5% (down-regulation) and 95% (up-regulation) quantiles and (b) a p-value threshold. In figure 2A only a p-value cut-off was used (as shown by the color coding). In Figure 3C, we again applied* *fold change and p-value thresholds (the fold change criterion of mass spectrometry data was set based on median +/- 2 * standard deviation). The figure legends have all been updated with the relevant information.*

The various Western blot panels shown throughout look convincing but the legends should indicate if these results were representative of replicate experiments.

*Thank you for this positive comment! The Western Blot panels depict representative experimental results of three or more biological replicates. The figure legends have been updated with the information.*

**Reviewer #2 (Remarks to the Author):**

The manuscript by Reich et al. seeks to understand the role of the endoplasmic reticulum unfolded protein response (UPR) in anti-folate resistance and metabolic reprogramming in astrocytoma. They suggest that chemical induction of the UPR controls a set of genes encoding enzymes that diverts metabolites from glycolysis to fuel mitochondrial folate‐based one‐carbon metabolism. Moreover, they suggest that the UPR contributes to chemoresistance of FDA approved folate-based anti-metabolites. Although the findings of the manuscript are original and interesting, there are major shortcoming in this manuscript. In particular further experimental evidence is required to support the title and claims in the manuscript. Please find suggested major and minor comments below:
Major comments

1. More than half of the main figures in the manuscript are focused on transcriptional, translational and proteomic analysis in LN308 astrocytoma cells after chemical induction of the UPR. Therefore, the title does not reflect the majority of the data in the manuscript. I suggest considering a revision of the title, as this manuscript mainly focuses on the kinetics of the transcriptome, translatome and proteome after chemical induction of the UPR, with minor focus on mitochondrial 1c metabolism and folate resistance.

*We agree with the reviewer about the fact that the title did not reflect the majority of the results in the old version of our study. We have therefore chosen a different title for the revised version of the manuscript, considering the criticism put forward by the reviewer:*

*‘****A multi-omics analysis reveals the Unfolded Protein Response regulon and a role of eIF2-phosphorylation in resistance to folate-based anti-metabolites’***

*We hope that this title adequately reflects the different aspects of our work presented in the manuscript.*

2. The studies are carried out in one cell line and confirmatory experiments should also be carried out in other cell lines.

*In the previous version of the manuscript, we presented data derived from experimentation on two different cell lines (LN308 and HEK293) that differ significantly in many characteristics (as outlined in the manuscript). We have now performed similar experiments in additional cell lines, with mostly similar results – part of the data are now included in the revised version of the manuscript (extended Fig 6) and the discussion has been extended accordingly.*

*We could identify only a single cell line that remains sensitive to treatment with MTX after induction of the UPR (K562 cells, see figure for the reviewer 1 below). We do, however, believe that inclusion of this data goes beyond the scope of the manuscript and we are currently addressing it in a follow-up study.*

***Figure for the reviewer 1:*** *K562 cells remain sensitive to treatment with MTX (concentrations indicated on the X-axis) after exposure to Tunicamycin. Experiments essentially performed as described in the manuscript.*

3. The choice of cell model is not clear. In the manuscript the authors have referred to breast cancer several times. Many tumor cells/ cell lines, including breast cancer cells lines have basal UPR activity. It would be interesting to see the effect basal UPR on mitochondrial 1C metabolism and reversal by inhibiting PERK activity.

*We are a bit puzzled by this comment as we refer to breast cancer only once and in the context of work performed by other groups that demonstrated that the induction of MTHFD2 results in a poor prognosis for patients (Liu et al., 2014; Nilsson et al., 2014).*

*To identify cells with ‘basal UPR activity’, we have screened numerous cancer cell lines for the expression of the UPR marker proteins ATF4, DDIT3 and XBP1 using our recently established, highly sensitive and quantitative targeted proteomics approach (Nguyen et al., Sci Rep 2019,* *A sensitive and simple targeted proteomics approach to quantify transcription factor and membrane proteins of the unfolded protein response pathway in glioblastoma cells). The results are depicted below in the figure for the reviewer 2. In non-stimulated cells (DMSO-treated, panels labelled Dm, select peptides and cell lines shown in figure below), expression of all three marker proteins is approx. 10-fold lower compared to Tunicamycin-treated cells (panels labelled Tm). Taken together, we could not identify a single cell line with a ‘basal UPR activity’ different from the HEK293 and LN308 cell lines (which are shown as a reference in the aforementioned publication) to assess its contribution to mitochondrial 1C metabolism or the effect of PERK inhibition in this context.*

***Figure for the reviewer 2:*** *Quantification of* *the cellular concentrations* *of the stress marker proteins ATF4 (left panels), DDIT3 (middle panels), and XBP1 (right panels) in various cell lines (as indicated in the legend on the right). Measurements were performed under normal growth conditions (top panels), or after induction of stress by administration of 2.5µg/ml of TM for 24h (lower panels). Please note the different scaling of the axes.*

4. The authors have not sufficiently shown that LN308 cells switch to mitochondrial 1C metabolism after UPR induction. Assessing the reliance of these cells for serine metabolism vs glycolysis should be examined. For example, monitoring proliferation of cells in serine depleted media after UPR activation/deletion of UPR components. Moreover, serine uptake and NADPH production in the mitochondria should be assessed after knockdown of PERK/p-eIF2α/ATF4 to link PERK directly to mitochondrial 1c pathway. Serine labelling can also demonstrate electron transfer through the mitochondrial 1c pathway.

*We are puzzled by the reviewer commenting on ‘the reliance of these cells for serine metabolism vs glycolysis’. Both pathways, in particular glycolysis, are considered to be highly active in malignantly transformed cells to sustain the high energy demand and anabolic requirements of the cell, driving proliferation and tumorigenesis. However, we thank the reviewer for the suggestion to analyze cell growth under conditions where proliferation is limited by the cells’ capacity to synthesize serine. When monitoring growth in serine-depleted medium we observe a modest reduction in cell proliferation (approx. 79% compared to the control). Strikingly, in the presence of 2.5µM TM the effect is less pronounced with 88% cell proliferation in the absence of Ser (relative to TM treatment only, see new figure S8). This supports the hypothesis that TM* *treatment can enhance the serine biosynthetic capacity through induction of serine biosynthetic enzymes, partially alleviating growth impairment in serine-depleted medium. The manuscript has been extended accordingly.*

*Furthermore, we have now included additional data in the manuscript that demonstrate increased flux of metabolites through the mitochondrial 1C metabolism after induction of the UPR (see new Fig 5B-E and S6). On the one hand, we demonstrate a significant change in the NADP^+^/NADPH ratio in stressed cells (Fig 5B), on the other hand we employ a stable isotope-labelled serine tracer for metabolic labeling and MS-based analyses to demonstrate a markedly elevated synthesis of glycine from serine and a significantly increased contribution of mitochondrial 1C metabolism-derived carbon units to deoxythymidylate nucleotide synthesis (Fig 5C-E and S&). In sum, these experiments support our initial findings and claims and directly demonstrate UPR-induced, enhanced metabolic flux through mitochondrial 1C metabolism.*

*The use of knockdown experiments to assess the contribution of PERK is discussed below in more detail (see bullet point #6).*

5. To support the in vitro experiments performed in figure 5, in vivo studies of astrocytoma cell models should be completed to examine tumour volume and UPR activation post anti-folate treatment to examine chemoresistance. PERK should be linked to anti-folate resistance by using PERK inhibitor or PERK knockout cells after folate treatment.

*We feel that these experiments go far beyond a typical revision, and – as interesting the potential findings might be – the proposed experiments should rather be part of a follow-up study.*

6. The experiment in figure 4A using pharmacological inhibitors should be further validated using siRNA/shRNA techniques against IRE1, PERK and ATF6.

*The inhibitors are well established in the field and we have experimentally addressed their specificity and efficacy (shown in Fig 4). Moreover, we see several complications associated with siRNA-mediated knockdown studies as (a) transfection by itself exerts stress on the cells, (b) RNAi treatment can result in pronounced off-target effects and can impact the expression of other proteins/mRNAs, (c) long incubation times are often required to achieve a sufficient knockdown of the individual proteins (depending on their turnover rates), and (d) lasting depletion/knockout of UPR factors itself has been reported to result in increased cell stress. For these reasons, we believe that in this particular case, the use of inhibitors is superior to RNAi-mediated knockdown, as it is rather immediate and does not involve transfection procedures. Moreover, in the case of IRE1, the inhibitor specifically targets its endonucleolytic activity while not affecting its kinase function, providing additional specificity that cannot be achieved by RNAi-based experimentation.*

*And finally – and most importantly –, we have confirmed by genetic means and independent of the use of inhibitors, that induction of the newly identified candidates is dependent on PERK-EIF2S1-ATF4 signaling (Figs 4B and 4C).*

*We believe that, even in the absence of RNAi-based experimentation, in sum we provide compelling evidence by numerous experimental approaches that induction of the aforementioned candidates is indeed mediated by the PERK-eIF2-ATF4 signaling pathway.*

7. The effect the authors attribute to UPR seems to be mediated by the PERK/eIF2 α/ATF4 pathway and this is a common pathway with ISR. The authors do not see a role for ATF6/IRE1 in their studies. As such, in line to what the authors have eluded to on page 15 and 16, ISR rather than UPR may be responsible for metabolic reprogramming. I believe that the authors need to make this distinction and to determine the role of other eIF2a kinases in the regulation of the pathway. Such data would significantly increase the impact of the study.

*We thank the reviewer for this insightful comment and have included additional data in the revised version of the manuscript. In the original version, we already exploited a phosphomimetic mutant of eIF2a to demonstrate that inhibition of eIF2B-mediated guanylyl nucleotide exchange in eIF2 and reduced formation of ternary complex can induce resistance to folate-based anti-metabolites in HEK293 cells.*

*We now additionally show that inhibition of EIF2S1 dephosphorylation (by the small molecule Salubrinal) has a similar effect in LN-308 cells (Fig S7). Moreover, we present additional data demonstrating that resistance to MTX treatment can also be induced by activation of the ISR kinase HRI (Fig 6D). This indeed demonstrates that not only PERK signaling during the UPR, but also signaling via other stress kinases (as part of the ISR) can induce resistance to MTX in cultured cells. We have extended the results and discussion sections of the manuscript accordingly.*

8. Do anti-folate compounds induce UPR/ISR?

*We do not detect significantly elevated expression levels of the stress-induced marker protein ATF4 after treatment for 6h or 16h with MTX (see figure below). After 24h a rather modest increase of ATF4 protein can be detected in MTX-treated cells relative to a control (see overexposed panel). The induction is, however, significantly weaker compared to cells that were treated with TM as a positive control.*

***Figure for the reviewer 4:*** *We have treated LN308 cells with 2.6nM MTX for 6h, 16h, or 24h. The chosen MTX concentration roughly equals the IC_50_ that we previously determined for LN308 cells, having a measurable, but modest impact on cell viability. Treatment with 2.5µM Tunicamycin served as positive-, DMSO as negative control. Cellular stress was evaluated by Western Blotting against ATF4 as a marker protein, GAPDH served as loading control.*

Minor comments

1. The qPCRs in Figure 4 are not well labelled, particularly the bottom left graph. It is also not clear from reading the figure legend what treatments were performed in this particular experiment. I also suggest changing the y-axis label to mRNA abundance/expression rather than mRNA induction.

*We thank the reviewer for pointing this out. We have changed the labelling of the graph – the label of the Y axis now reads RNA abundance change (log2 FC). Moreover, as suggested by the reviewer, we have revised the figure legend to better describe the treatments performed in this set of experiments.*

2. Some figure legends describe the results rather than describing the experimental information, for example figure 4 ‘Expression of a phosphomimetic eIF2a protein (lanes S51D), but not a non‐phophorylatable variant (lanes S51A), increases SLFN5, SHMT2, and MTHFD2 mRNA abundance in HEK293 cells’

*We have changed the figure legends according to the reviewer’s recommendation and in line with the policy of Nature Communications.*

3. Does the treatment with TM and TH induce cell death? What was % cell death at each sampling time point?

*As described in the manuscript, we have chosen concentrations of TH and TM that result in only a modest decrease in cell viability and proliferation as to be able to analyze stressed but otherwise ‘healthy’ cells. Exemplarily, cell viability after 24h of treatment with 2.5µg/ml of TM is at approx. 85% (as measured by MTS assay, see figure for the reviewer 5 below)*

***Figure for the reviewer 5: A:*** *Survival of LN308 cells in DMEM medium after 24h of continuous treatment with 2.5µg/ml TM relative to a control treatment with DMSO. Cell viability was assessed by an MTS assay, displayed is a mean value ±SD of 6 biological replicates.* ***B:*** *Survival of LN308 cells in MEM medium in the presence (orange/yellow bars) or absence of serine (green bars). Cells were either treated with 2.5µM TM (light bars) or DMSO as a control (dark bars). Assay as described for panel A. depicted are mean values ±SD of 8 technical replicates.*

**Reviewer #3 (Remarks to the Author):**

This manuscript reports on cellular stress response pathways that modify responses to chemotherapeutic drugs. Deep sequencing analysis of transcription and translation are combined with proteomics in order to characterize short- and long-term responses to mechanistically distinct inducers of the ER UPR. Joint analysis of these data reveal a core UPR regulon that includes known targets of this well-studied pathway as well as the complete pathway for folate-based 1-carbon metabolism. Notably, folate is a target for anti-cancer chemotherapy, and UPR induction provides dramatic resistance to folate antimetabolite drugs.

This study uncovers additional targets of the UPR / integrated stress response pathway and demonstrates a clear impact on cell metabolism and physiology with likely clinical relevance. As such, it is likely to attract broad interest. Overall the data are clear and support the key conclusions, though I have specific concerns with some interpretations as described below.

1. Ribosome profiling and proteomic mass spectrometry data are used to argue for a general reduction in translation. For example, at the top of p. 7 it is written that “we find…an overall reduction from RPFs from numerous loci…” and on p. 8 that proteomics results are “…indicative of a globally reduced protein synthesis rate.” However, both approaches suffer from a global normalization problem that is not addressed in this manuscript. A ribosome profiling experiment reports on the overall pool of translating ribosomes and is insensitive to changes in the bulk number of translating ribosomes (e.g., Liu et al., Cell Systems 2017). Likewise, the mass spectrometry data captures the fractional abundance of individual proteins in the overall proteome. Neither approach can directly assess changes in bulk translation without careful use of spike-in standards.

Alternate techniques, such as pulse labeling with OP-Puro or L-AHA permit analysis of bulk protein synthesis.

*We thank the reviewer for this insightful comment. Indeed ribosome profiling experiments usually do not reflect changes to global translation rates. We had already performed metabolic labelling analyses and polysome analyses to monitor translation rates more directly but we did not present the data - a shortcoming that we now corrected in the revised version of the manuscript. In brief, by quantification of ^35^S Methionine incorporation we directly demonstrate a reduction of protein synthesis rates in TM treated cells by approx. 35% (new Fig S3A). Moreover, by gradient fractionation of ribosomal complexes, we show a reduction of polysomes (in particular of heavy polysomes) after treatment with TM (new Fig S3B).*

2. The manuscript cites Andreev et al., eLife 2015 in discussing ISR translation, but not Sidrauski et al., eLife 2015, which seems at least as relevant.

*Thanks for pointing out this blunder! We have now cited Sidrauski et al. in our manuscript.*

3. The manuscript should compare ISR translation targets with those reported in Andreev et al. or in Sidrauski et al. Sidrauski et al. use tunicamycin, one of the UPR inducers here, whereas Andreev et al. use arsenite; each comparison would add to the analysis.

*Surprisingly, we find only a very limited overlap between the individual datasets. The most likely explanation for this discrepancy is that treatment conditions differ between the three studies. Our analyses focus on time points 2h and 6h after induction of stress, whereas Andreev et al. and Sidrauski et al. analyze translation at earlier stages, 30min and 1h after treatment, respectively. Moreover, different compounds, or concentrations thereof, are used to induce cellular stress: Andreev et al. use 40µM arsenite, Sidrauski et al use 1µM TM, and we use 2.5µM TM. Finally, Andreev et al. and Sidrauski et al. analyzed translation in HEK293 cells while our study was performed in LN-308 cells. Combined these differences might explain the limited overlap of the datasets.*

*We are currently performing additional experiments to better understand translational regulation under various stress conditions in different cellular models. In agreement with Sidrauski et al. and Andreev et al., we observe robust induction of ATF4 translation in HEK293 cells after 2h of TM treatment (as measured by ribosome profiling), however, in LN308 cells this effect is less pronounced and we do not reach statistical significance regarding the induction of ATF4 translation in our ribosome profiling data. However, our data are rather preliminary and we feel that they would dilute the focus of the present manuscript, if included.*

4. The MTX resistance data are quite striking, but tests linking this to the ISR are performed in a different cell line (HEK293). It seems important to show that MTX resistance depends on UPR / ISR signaling specifically in transformed cells. Furthermore, the drugs ISRIB and salubrinal provide a straightforward way to manipulate the ISR that can be applied in many different cell types without transfection or transduction. Furthermore, results using these drugs can translate clinically in a way that transgenic expression of phosphomimetic proteins cannot.

*We thank the reviewer for bringing this to our attention! We have now included additional data in the manuscript that experimentally demonstrate a role of UPR/ISR signaling specifically in the malignantly transformed cell line LN308. On the one hand, the small molecule compound BTdCPU is used for activation of the EIF2S1 kinase HRI (Fig 6D), on the other hand, we employed Salubrinal to inhibit dephosphorylation of EIF2S1 by PP1 (new Fig S7). In both cases we observe increased resistance to MTX treatment.*

*Unfortunately, however, so far, we could not establish conditions under which administration of ISRIB to stressed cells resulted in re-sensitization to treatment with MTX. By Western Blotting we can demonstrate that ISRIB prevents accumulation of ATF4 at early time points after administration of TM (up to approx. 4h, data not shown). However, at later stages, and once the EIF2S1 phosphorylation levels exceed a certain threshold, ISRIB is not effective any more, resulting in strong accumulation of ATF4 (as previously published by Rabouw et al., 2019). This might explain why resistance to MTX can still be observed in ISRIB-treated cells upon administration of TM.*

5. The IC50 is 5FU is said to remain unaffected (p. 14 and Figs 5B and C) after UPR induction, but it seems there is actually a modest but real increase in resistance.

*We have performed a two-sided student’s T-test, however, the P-values do not reach the required level of significance to claim that there is indeed an increase in resistance (values are just above 0.05).*

6. as a minor point, at the top of p. 12, “or” in the list of ATF4-responsive genes should be “and”.

*Thanks for pointing this out!*

**Reviewer comments, second version:**

Reviewer #1 (Remarks to the Author):

The authors have addressed my concerns in their detailed revision.

Thomas Preiss.


Reviewer #2 (Remarks to the Author):

The authors have address my comments/concern satisfactorily. I'd like to congratulate the authors and commend them on the effort they have put into revising their manuscript.


Reviewer #3 (Remarks to the Author):

Revisions have addressed my major concerns with the initial submission. The analysis of bulk translation (by polysome profiling) and protein synthesis (by analogue metabolic labeling) clarify questions related to global normalization. Demonstration of MTX resistance in LN308 cells with a range of UPR inducers show that the induction of 1C metabolism and drug resistance is a general effect that could arise in tumors. The new metabolomic data also strengthens this argument.

One (hopefully minor) concern regards the legend of Fig. 4C -- was this data from 293 cells or LN308 cells?

**Author rebuttal, second version:**

**Reviewer #1 (Remarks to the Author):**

The authors have addressed my concerns in their detailed revision. Thomas Preiss.

**Reviewer #2 (Remarks to the Author):**

The authors have address my comments/concern satisfactorily. I'd like to congratulate the authors and commend them on the effort they have put into revising their manuscript.

**Reviewer #3 (Remarks to the Author):**

Revisions have addressed my major concerns with the initial submission. The analysis of bulk translation (by polysome profiling) and protein synthesis (by analogue metabolic labeling) clarify questions related to global normalization. Demonstration of MTX resistance in LN308 cells with a range of UPR inducers show that the induction of 1C metabolism and drug resistance is a general effect that could arise in tumors. The new metabolomic data also strengthens this argument.

One (hopefully minor) concern regards the legend of Fig. 4C -- was this data from 293 cells or LN308 cells?

Thanks for pointing this out! The experiment has been performed in HEK293 cells (the figure legend has been corrected).

**Reviewer comments, third version:**

**Author rebuttal, third version:**
